# Kinetics and Reaction Mechanism of Biothiols Involved in S_N_Ar Reactions: An Experimental Study

**DOI:** 10.3389/fchem.2022.854918

**Published:** 2022-06-08

**Authors:** Paola R. Campodónico, Jazmín Alarcón-Espósito, Belén Olivares

**Affiliations:** ^1^ Centro de Química Médica, Instituto de Ciencias e Innovación en Medicina, Facultad de Medicina, Clínica Alemana Universidad del Desarrollo, Santiago, Chile; ^2^ Departamento de Química Orgánica y Fisicoquímica, Facultad de Ciencias Químicas y Farmacéuticas, Universidad de Chile, Santiago, Chile

**Keywords:** S_N_Ar reactions, reaction mechanism, border mechanisms, biothiols, reactivity patterns

## Abstract

Few kinetic parameters, or reaction rates, are known up to date in detail about 1-chloro and 1-fluoro-2,4-dinitrobenzene (ClDNB and FDNB, respectively) with a series of biothiols in aqueous media. These biological nucleophiles with thiol groups have been widely used as a reference in nucleophile reactivity assays due to their prevalence and cellular abundance. The main aim of this study was to elucidate the reaction mechanism based on Brönsted-type plots and reactivity patterns of the electrophile/nucleophile pairs. A complete kinetic study was performed in terms of the comparison of Brönsted-type slope parameters (*β*_nuc_) for the reactions and was used for assigning the mechanism and the rate-determining step associated with the reaction route. A mass spectrometry analysis demonstrated that the nucleophilic center of the biothiols is the -SH group and there is only one kinetic product. The kinetic study suggests that the reaction mechanism might be the borderline between concerted and stepwise pathways. An amine–enol equilibrium for the most reactive nucleophiles appears to be the main determining factor controlling the nucleophilic attack in the nucleophilic aromatic substitution reactions investigated, highlighting the anionic form for these nucleophiles. This amine–enol equilibrium involves a hydrogen bond which stabilizes the intermediate species in the reaction pathway. Thus, intramolecular bonds are formed and enhance the nucleophilic strength through the contribution of the solvent surrounding the electrophile/nucleophile pairs. Finally, we highlight the importance of the formation of electrophile/nucleophile adducts that could modify structures and/or functions of biological systems with potential toxic effects. Therefore, it is essential to know all these kinetic and reactivity patterns and their incidence on other studies.

## Introduction

Electrophiles are often potential substrates that develop adducts in a critical step of pathogenic processes, which are initiated by the exposure of these chemicals to biological nucleophiles (Aptula et al., 2005; Schultz et al., 2006; Campodónico and Contreras, 2008). The reactions between electrophiles and biological nucleophiles have early been studied by Coles, who hypothesized that the reactions of these species could have toxic effects by the formation of electrophile/nucleophile (E^+^/Nu) adducts and modify structures and/or functions of proteins, deoxyribonucleic acid (DNA), or ribonucleic acid (RNA) (Coles, 1984; LoPachin et al., 2009). Electrophilicity and nucleophilicity concepts are based on the general acid–base theory of Brönsted and Lowry (Lowry, 1923) and the valence electron theory of Lewis (Lewis, 1923), where E^+^ and Nu^−^ correspond to electron-deficient and electron-rich species (Ingold, 1929; Ingold, 1933; Ingold, 1934). The activities of substrates and biological targets depend on the reactivity patterns of E^+^/Nu^−^ pairs and their reaction mechanisms (Carlson, 1990). The most recurrent reactions of these E^+^/Nu^−^ pairs correspond to Michael reactions, nucleophilic substitutions (NS), and nucleophilic aromatic substitutions (S_N_Ar) among others (Aptula et al., 2005; Schultz et al., 2006). Biological nucleophiles such as biothiols are involved in many cellular functions and human diseases (Seshadri et al., 2002). These molecules have a thiol (–SH) group in their chemical structure. The most known biothiol is the tripeptide glutathione (GSH). Despite the importance of biological processes involving biothiols, only fragments of fundamental physical–chemical aspects are well understood.

**FIGURE 1:**
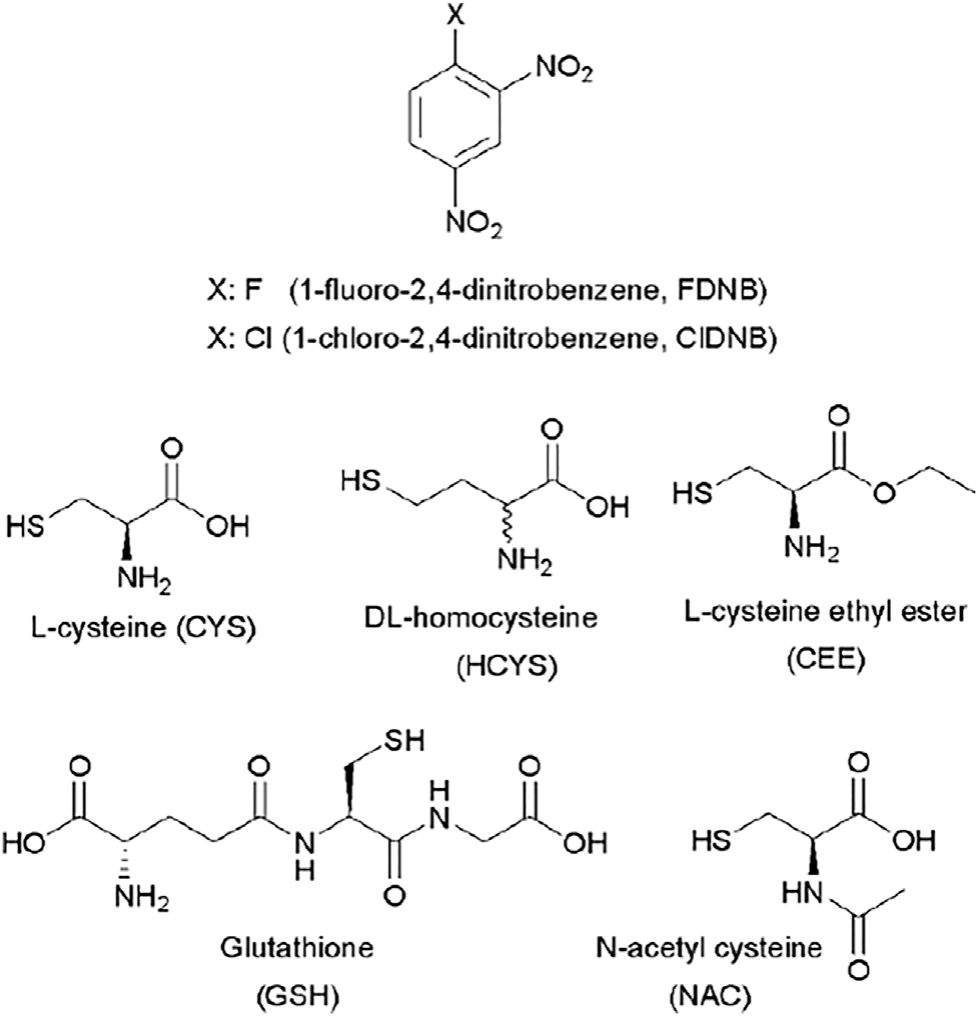
Chemical structures of substrates and biothiols used in this work.

In order to investigate one of these types of reactions (S_N_Ar), the main aim of this work was to show that kinetic studies can be used to better understand the mechanism which is derived from reactions of known substrates: 1-chloro and 1-fluoro-2,4-dinitrobenzene (ClDNB and FDNB, respectively) with a series of biothiols in aqueous media (see Figure 1) (Ormazábal-Toledo et al., 2013a; Alarcón-Espósito et al., 2015, 2017; Sánchez et al., 2018a, 2018b). Biological nucleophiles with the thiol group have been widely used as a reference in nucleophile reactivity assays due to their prevalence and cellular abundance (Roberts et al., 2007; Schwöbel et al., 2011). ClDNB and FDNB compounds are classified by the structural alert (SA) such as i) SA_27 (nitro aromatic) and ii) SA_31a (halogenated benzene) in the compilation of chemical linked to carcinogenicity and mutagenicity (Ashby and Tennant, 1988; Worth et al., 2007; Benigni and Bossa, 2011). However, only few kinetic parameters or reaction rates for these systems are known in detail. In this study, kinetic results are discussed in terms of the comparison of Brönsted-type slope parameters ( $\beta_{nuc}$ ) for the reactions and will be used for assigning the mechanism and rate-determining step (RDS) (Newington et al., 2007; Um et al., 2007; Ormazábal-Toledo et al., 2013a, 2013b; Gallardo-Fuentes et al., 2014; Alarcón-Espósito et al., 2015; Campodónico et al., 2022). Figure 1 shows chemical structures and acronyms of substrates and biothiols used in this work.

## Materials and Methods

### Materials

1-Chloro and 1-fluoro-2,4-dinitrobenzene and all the biothiols were of the highest quality available such as commercial products by Merck and Sigma Aldrich. The certificate of analysis guarantees purity was ≥99%.

### Kinetic Measurements

The kinetics of the reactions were performed spectrophotometrically (*λ* = 336 nm) using a diode array spectrophotometer in aqueous and buffer phosphate solutions at 25.0 and 37.0 ± 0.1°C, ionic strength 0.2 M (KCl) for aqueous media at three different pH values maintained by partial protonation of the biothiols (pH = $pK_{a}$ and pH = $pK_{a}\;$ ± 0.3). Thus, equilibrium between the free biothiol as the thiolate group and its protonated form (thiol group) was established. All the reactions were studied under excess nucleophiles over substrates (at least 10 times greater than the substrate concentration) in order to establish the pseudo-first-order kinetics. The kinetic study started by injecting the substrate stock solution in acetonitrile (10 μL, 0.01 M) into the biothiol solution (2.5 ml in the spectrophotometric cell). The formation of the colored kinetic product was monitored by UV-vis spectroscopy. In all the runs, the pseudo-first-order rate coefficients ( $k_{obs}$ ) were found for all reactions. The $k_{obs}$ values were determined by means of the spectrophotometric kinetic software for first-order reactions at the wavelength corresponding to the kinetic product. Note that, the measurements at pH = $pK_{a}$ and 0.3 units up and down were performed in order to determine the possibility of acid and/or basic catalysis by the media. Then, the relationships between $k_{obs}$ *vs* [B] (concentration of biothiols) should be straight lines or straight lines with smooth deviations, which will discard a catalysis processes by the media. The $k_{N}$ values are obtained from plots in accordance with Eq. 1:

$$k_{obs}=k_{0}+k_{N}\left[\text{B}\right],$$ (1)

where $k_{0}$ and $k_{N}$ are the rate coefficients for solvolysis and nucleophilic attack of the substrate, respectively. These values were obtained as the intercept ( $k_{o})$ and slope ( $k_{N})$ of linear plots for the reactions between the substrate with each biothiol at different concentrations, denoted by [B] . See more details in the Supplementary Material. This kinetic value was taken from previous kinetic studies cited in the reference section and previous works performed by our group. (Castro, 1999; Um et al., 2007; Ormazábal-Toledo et al., 2013b; Gallardo-Fuentes et al., 2014; Alarcón-Espósito et al., 2015 and 2017; Campodónico et al., 2020 and 2022).

### Mass Spectrometry

This was operated in a negative mode. Accurate mass spectra were recorded from 100 to 550 m/z. For the fragmentation study, a data-dependent scan was performed using the electrospray ionization mode with an AB Sciex Triple Quad 4500 mode. A computer was equipped with Analyst software, version 1.6.2, handled data analysis. The compounds from the reaction between substrate ClDNB and the nucleophiles: GSH and S-methyl glutathione (Me–GSH) were identified by their corresponding spectral characteristics, accurate mass, mass spectra, and feature fragmentation. This analysis supports the existence of a kinetic product at 474 m/z followed at 336 nm in a negative mode. So, from a mass spectrometry analysis it is possible to assign one chemical structure to each m/z ratio. It is worth noting that the reaction products for reactions between ClDNB and FDNB with biothiol series will be the same or similar. Hence, in this analysis only ClDNB was considered.

**FIGURE 2:**
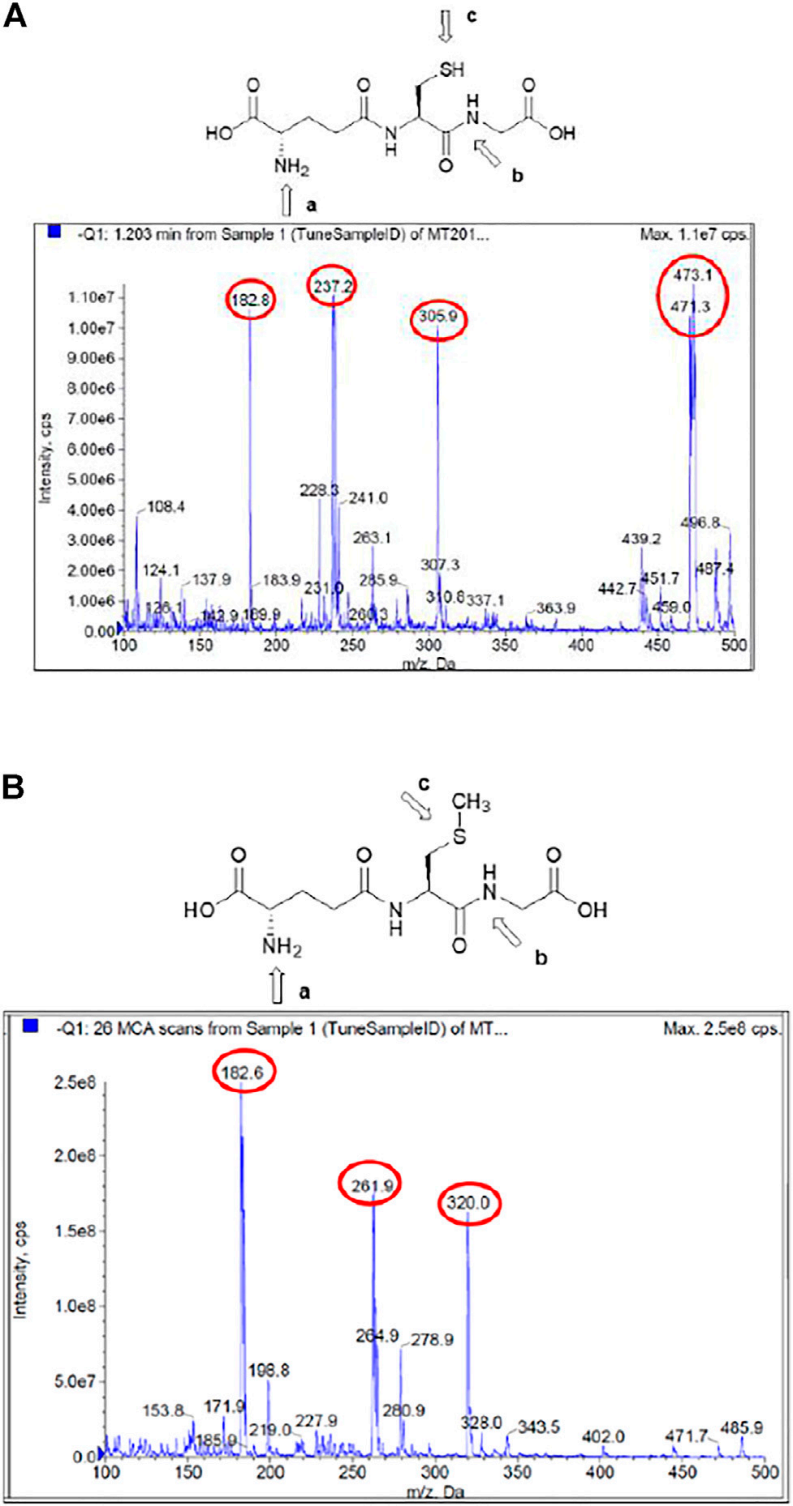
**(A)** Mass/charge ratio for the reaction between GSH with ClDNB in aqueous media. **(B)** Mass/charge ratio for the reaction between Me–GSH with ClDNB in aqueous media.

**FIGURE 3:**
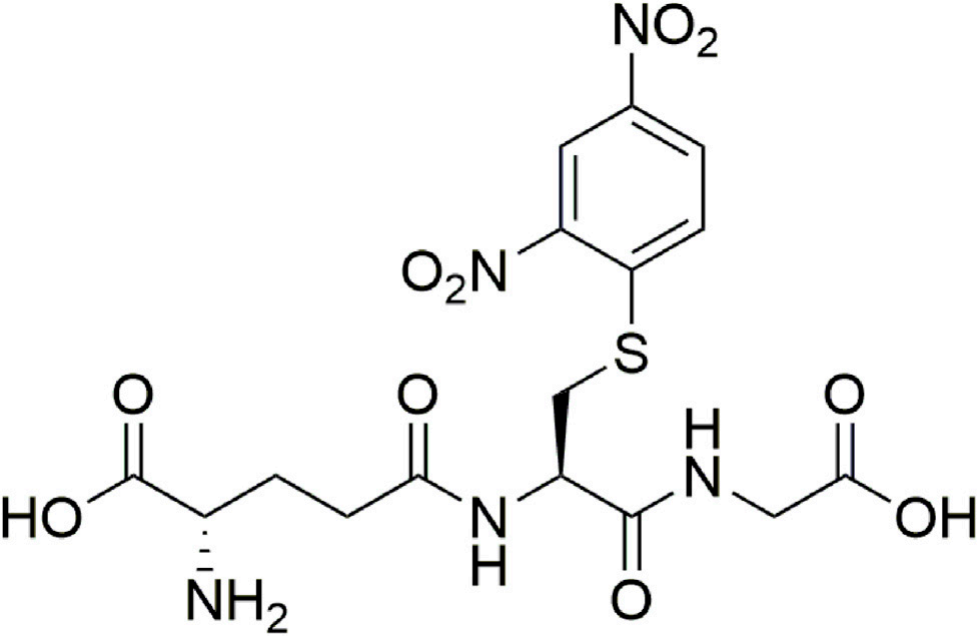
Chemical structure associated with mass/charge ratio for the reaction product between GSH and ClDNB.

Figure 2A considers the fragmentation patterns associated with the reaction between GSH with ClDNB. The chemical structure of GSH in Figure 2A shows the possible nucleophilic centers located on N- (***a***, ***b***) and S- (***c***) groups denoted by arrows. Note that, this is a general chemical structure of GSH, and it does not consider the protonation states. Figure 2A shows four strong signals: 182.8, 237, 305.9, and 473. One of the most important signals corresponds to m/z = 473, which was assigned to the kinetic product (see Figure 3). The fluctuation of m/z between 471 and 473 could be attributed to different protonation states of the kinetic product. However, the reaction product may be oriented toward those three positions (***a***, ***b, or c***), but position ***b*** might be discarded by steric hindrance. However, the most important reason is the chemical nature of ***b*** position; it is an amide, which is a weak nucleophilic center due to resonance effects with the carbonyl group. Thus, the possibilities of nucleophilic attack should be ***a*** and ***c*** oriented to SH- or amino (NH_2_-) groups in the chemical structure of GSH. In order to determine the reaction center in GHS, the mass analysis of the reaction between Me–GSH with ClDNB (see Figure 2B below) was performed. In contrast, Figure 2B shows no signal attributed to the kinetic product (m/z = 473). The chemical structure of Me–GSH in Figure 2B shows it is blocked in the ***c*** position by a methyl group. Therefore, the only nucleophilic center on GHS able to react with ClDNB will be ***c*** position, the SH- group. (See Figures 2A, 3).

### Product Analysis

Product authentication was performed by a complete mass spectroscopy analysis which suggested the presence of a series of compounds from the reacting pair in aqueous media. Figure 2 shows the mass spectrum for the reaction between ClDNB and GHS (Figure 2A) and Me–GSH (Figure 2B), respectively.

**FIGURE 4:**
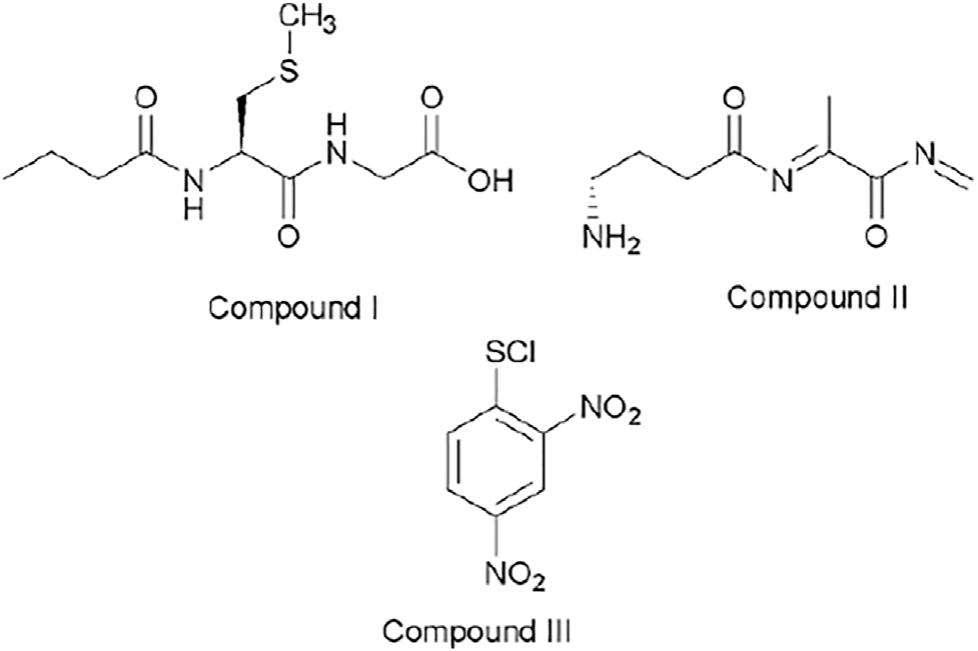
Chemical structures associated with mass/charge ratio for the reaction between ClDNB and GSH and Me–GSH.

Figure 4 shows the possible compounds derived from the studied reactions. Figure 2A shows another three strong signals: 182.8, 237.0, and 305.9 and Figure 2B shows three strong signals: 182.8, 261.9, and 320.0. The signals at 305.9 (Figure 2A) and 320.0 (Figure 2B) might be attributed to GSH and Me–GSH, respectively. As reaction conditions are pseudo-first-order, the concentration of nucleophile is almost 10 times more concentrated in comparison to the substrate. Figure 2A shows a signal located at 237.0, which is not shown in Figure 2B. At the same time, Figure 2B shows a signal located at 261.9, which is not shown in Figure 2A. So, these signals might be attributed to decomposition products from the reacting pair where the signal located at 237.0 is attributed to compound III and the signal located at 261.9 to compound I. Finally, both spectra have only one common signal located at 182.8. This might be associated to a decomposition product from the GHS and/or Me–GSH named compound II.

## Results and Discussion

Under the experimental conditions used, only one product formation was spectrophotometrically observed for all the reactions which displayed an increase of a band centered in the range of 330–550 nm and was attributed to the corresponding reaction product for all nucleophiles studied (see Figure 3). Therefore, the possibility of a nucleophilic attack at the unsubstituted ring positions of the substrate is discarded (Um et al., 2007; Gabsi et al., 2018).

**FIGURE 5:**
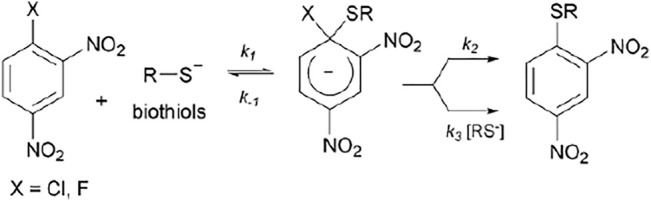
General reaction mechanism for a S_N_Ar between 1-halogen-2,4-dinitrobenzene with a biothiol in the anionic form.

The S_N_Ar process is well documented in the literature as a stepwise mechanism ( $S_{N}Ar_{stpw}$ ) (Crampton et al., 2004, 2006; Um et al., 2007; Campodónico et al., 2020, 2022). Figure 5 shows this mechanistic route, where the first step leads to the formation of a zwitterionic complex, namely, the Meisenheimer complex (MC), for which two processes have been postulated regarding protonated nucleophiles: *i*) expulsion of the leaving group (LG) followed by the fast proton loss to give the reaction product ( $k_{2}$ ) and *ii*) the base-catalyzed deprotonation of the zwitterionic complex ( $k_{3}$ ) that loses the halogen atom to give the reaction product (Ormazábal-Toledo et al., 2013a; Alarcón-Espósito et al., 2015, 2017; Sánchez et al., 2018a, 2018b). It is worth noting that biothiols under our experimental conditions might be in an anionic form, thus Figure 5 shows that the catalyzed pathway ( $k_{3}$ route) may be discarded from Figure 5 and the reaction mechanism for E^+^/Nu^−^ pairs should be shown as *i*) the formation of the MC and *ii*) the expulsion of the LG to give the reaction product (Banjoko and Babatunde, 2004; Um et al., 2007; Ormazábal-Toledo et al., 2013a; Terrier, 2013; Gazitúa et al., 2014; Mortier, 2015; Alarcón-Espósito et al., 2017; Sánchez et al., 2018a, 2018b).

Considering the established $S_{N}Ar_{stpw}$ mechanism for these reactions, the kinetic analysis shows that the pseudo-first-order rate constant for the studied reactions can be expressed as Eq. 2. It was derived applying steady-state approximation for the S_N_Ar process (see the Supplementary Material for more details).

$$k_{obs}=\;\frac{\left(k_{1}k_{2}\left[\text{B}\right]+\;k_{1}k_{3}{\left[\text{B}\right]}^{2}\right)}{k_{1}+\;k_{2}+\;k_{3}\left[\text{B}\right]}.$$ (2)

Note that, the $\;k_{obs}$ values were experimentally obtained at different concentrations of free biothiol ( ${\left[\text{B}\right]}_{F}$ ) for each pH value in aqueous media, respectively. These results were plotted using $k_{obs}\;vs.\;{\left[\text{B}\right]}_{F}\;$ in order to obtain the $k_{N}$ values for each biothiols studied (see Table 1 and kinetic measurements section). All linear plots passed through the origin, suggesting the contribution of the solvent to the values $k_{obs}\;$ is negligible (Um et al., 2007). On the other hand, all the plots between $k_{obs}\;vs.\;{\left[\text{B}\right]}_{F}\;$ are shown to be straight lines (see Supplementary Figures S1–S24) discarding a catalyzed pathway by a second molecule of nucleophile ( $k_{3}$ route). Thus, $k_{obs}\;$ values can be expressed as Eq. 3, where the $k_{N}\;$ rate coefficients are determined from the slope of the linear plots (see Equation 1), where $k_{-1}+\;k_{2}\gg >k_{3}\left[B\right]$ . See Supplementary Tables S1–S24 and Supplementary Figures S1–S24. Here, ${\left[\text{B}\right]}_{F}$ is denoted by ${\left[\text{Nu}\right]}_{F}$ , and Nu corresponds to the nucleophile, specifically the concentration of nucleophile.

$$k_{obs}=\;k_{N}\left[\text{B}\right],\;where\;k_{N}=\;\frac{k_{1}k_{2}}{\left(k_{-1}\;+\;k_{2}\right)}.$$ (3)

**TABLE 1:** Nucleophilic rate constant values for the reaction between ClDNB with biothiol series in aqueous media and phosphate solution at 25°C and 37°C.

Biothiol compound	$pK_{a}$	ClDNB aqueous media		ClDNB buffer phosphate	
		$k_{N}$ (sM)^−1^ 25°C	$k_{N}$ (sM)^−1^ 37°C	$k_{N}$ (sM)^−1^ 25°C	$k_{N}$ (sM)^−1^ 37°C
L-Cysteine ethyl ester	6.50	0.10 ± 3 × 10^−3^	0.18 ± 6 × 10^−3^	0.13 ± 5 × 10^−3^	0.23 ± 0.01
Cysteine	8.10	0.12 ± 6 × 10^−3^	0.72 ± 0.02	0.15 ± 0.01	0.41 ± 0.02
DL-Homocysteine	8.25	0.25 ± 8 × 10^−3^	0.48 ± 0.01	0.57 ± 0.01	1.75 ± 0.04
Glutathione	8.75	1.26 ± 0.05	2.16 ± 0.07	1.74 ± 0.04	3.35 ± 0.10
N-Acetylcysteine	9.50	1.88 ± 0.08	3.80 ± 0.13	2.12 ± 0.05	5.14 ± 0.14

The $k_{N}$ and $pK_{a}$ values are summarized in Tables 1, 2 for both substrates (kinetic details in the Materials and methods section and the Supplementary Material). Data for ClDNB were measured in aqueous media and buffer phosphate media at 25°C and 37°C (see Table 1). In contrast, FDNB (see Table 2) only considered measurements in aqueous media at 25°C. For the studied reactions, the $k_{N}$ values, as well as those for the $\;pK_{a}\;$ of conjugate acids of thiols were statistically corrected with *q* = 2 and *p* = 1. Parameter *q* is the number of equivalent basic sites in the thiolate and *p* is the number of equivalent dissociable protons of the thiol (Bell, 1973; Thomas, 1974). The value accompanying $k_{N}$ coefficients correspond to the error associated with the slope to obtain these values.

**TABLE 2:** Nucleophilic rate constant values for the reaction between FDNB with biothiol series in aqueous media at 25°C.

Biothiol	$pK_{a}$	FDNB $k_{N}$ (sM)^−1^
L-Cysteine ethyl ester	6.50	5.45 ± 0.17
Cysteine	8.10	21.32 ± 0.66
Glutathione	8.75	66.90 ± 1.62
N-Acetylcysteine	9.50	95.63 ± 3.28

Another mechanistic route might be a concerted pathway ( $S_{N}Ar_{Conc}$ ). However, up to date there are some reports about concerted mechanisms on S_N_Ar reactions (Jencks and Gilchrist, 1968; Banjoko and Babatunde, 2004; Terrier, 2013; Um et al., 2014; Neumann et al., 2016; Neumann and Ritter, 2017; Gazitúa et al., 2014; Kwan et al., 2018; Gallardo-Fuentes and Ormazábal-Toledo, 2019; Campodónico et al., 2020, 2022). In this case, the nucleophilic attack and LG departure occur at the same time without MC formation (Jencks and Gilchrist, 1968).

**FIGURE 6:**
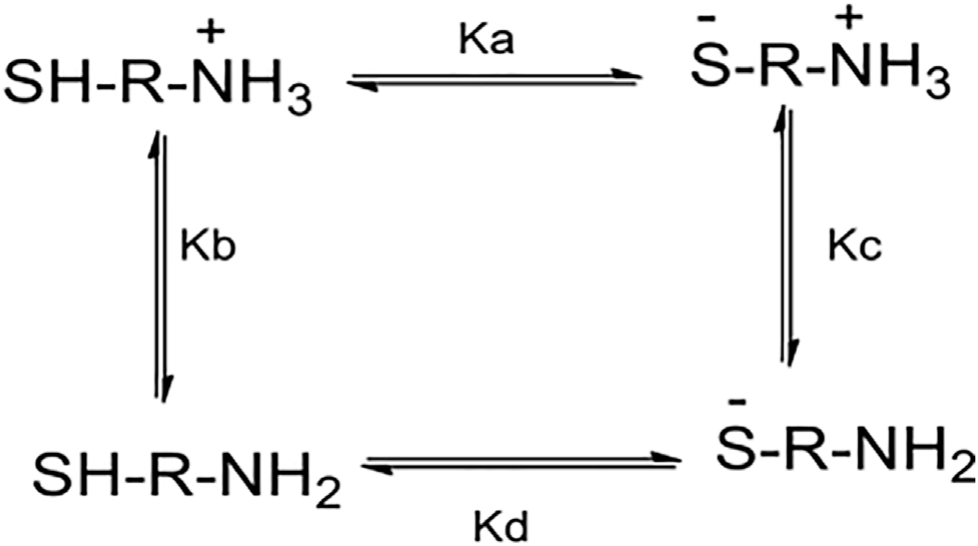
Acid–base equilibria related to general aminothiol in the pH range between 6 and 12. $K_{a\;}$ – $K_{d}$ are the macroscopic constants for each equilibrium (Sardi et al., 2013; Calfumán et al., 2017).

A preliminary inspection of Tables 1, 2 shows that reactivity patterns of the nucleophiles in aqueous media as reaction media toward ClDNB and FDNB increased in the following order: *N-acetyl cysteine > Glutathione > Cysteine > L-cysteine ethyl ester*. This order agrees with the basicity of the sulfhydryl group in biothiol ( $pK_{a}$ values). The only exception was for homocysteine at 37°C, which might be attributed to the major long chain (two carbon atoms) of the alkyl chain separating the sulfhydryl group in the amino group promoting the freedom of the nucleophilic center (see Figure 1). Similar results have been reported about steric hindrance of biothiols toward 1,4-addition reactions and coumarin derivatives (García-Beltrán et al., 2011, 2015). Other contributing factors to the nucleophilicity power of biothiol are polarizability, desolvation (Lin et al., 2009; Yuan et al., 2011), and the reaction media among others (Sardi et al., 2013; Calfumán et al., 2017; Glossman-Mitnik and Maciejewska, 2020). Sardi et al. (2013) reported the acid–base equilibria related to a general aminothiol in the pH range between 6 and 12, see Figure 6 below (Sardi et al., 2013). For instance, Benesch et al. (1955) early reported the macroscopic constants for each equilibrium ( $K_{a\;}$ – $K_{d}$ ) of L-cysteine and L-cysteine ethyl ester, two biothiols used in this study. (Benesch et al., 1955). These values are shown in Table 3 (below).

**TABLE 3:** Macroscopic constants for each equilibrium ( $K_{a\;}$ – $K_{d}$ ) denoted in Figure 6 for L-cysteine and L-cysteine ethyl ester.

Biothiol	pKa	pKb	pKc	pKd
L-Cysteine	8.53	8.86	10.36	10.03
L-Cysteine ethyl ester	7.45	6.77	8.41	9.09

The following analysis is based on the kinetic response and its possible relationships with the macroscopic constants. Then, the most nucleophilic biothiols toward the substrates correspond to N-acetylcysteine (see Tables 1, 2 in the text) suggesting that $K_{c}$ (in Figure 6) shifts toward the anionic form (^−^S-R-NH_2_, in Figure 6). Hence, considering N-acetylcysteine compound as a reference, the reactivity was analyzed. Glutathione is 1.5 times less reactive locating the compound in the same equilibria as an anionic species. DL-Homocysteine compound is approximately 7,500 times less reactive, suggesting it is located in $K_{d}$ equilibria close to the neutral species (HS–R–NH_2_, in Figure 6). Finally, cysteine and L-cysteine ethyl ester compounds should be located in $K_{b}$ . These compounds are 16,000 and 19,000 times less reactive than N-acetylcysteine suggesting the amino protonated forms (HS–R–NH_3_^+^, in Figure 6). Note that, compounds that contain their chemical structures, sulfhydryl and ammonium groups, have been early studied. (Benesch, et al., 1955). These compounds have three dissociable protons, and the carboxyl group at low pH values will be fully ionized and the other protons belong to -SH and NH_2_- groups (see Figure 6). Then, $pK_{a}$ values reported in Tables 1, 2 correspond to the -SH group, because this group is considered more reactive than the NH_2_- group toward the substrates. This fact was reinforced by the product analysis (see Material and methods section). Considering the values reported in Table 3 for cysteine and L-cysteine ethyl ester compounds and the pH values under the experimental conditions (see the Materials and methods section and Tables 1, 2), it is possible to analyze the relationship between the free biothiol as the thiolate group and its protonated form (thiol group), suggesting that the predominant species should be SH–R–NH_3_^+^/SH–R–NH_2_ ( $K_{a}\;and\;K_{b},\;respectively$ ). The results agree with the kinetic analyses. On the other hand, the relationships between the macroscopic constant (Figure 6 and Table 3) suggest for both biothiols that $K_{a}$ (in Figure 6) shifts toward the protonated form.

**FIGURE 7:**
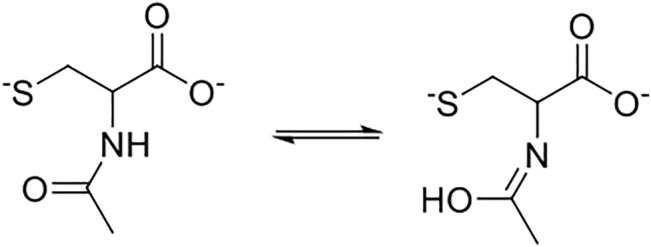
Possible tautomeric equilibrium for N-acetylcysteine and glutathione compounds.

Note that the most reactive nucleophiles (N-acetylcysteine and glutathione) have an amide (R_2_-N-(CO)-R) group in their chemical structures, which might establish amine–enol equilibrium (see Figure 7 below). Then, the tautomeric equilibrium may be stabilizing the thiolate form enhancing their reactivities (see Figure 7). On the other hand, homocysteine, cysteine, and L-cysteine ethyl ester compounds cannot establish the amine–enol equilibrium mentioned before, which reinforces it has a key role in the reactivity patterns (see Tables 1, 2 in the text and see Figure 1) for N-acetylcysteine and glutathione. Therefore, the amine–enol equilibrium appears as the main determining factor controlling the nucleophilic attack in a S_N_Ar reaction. The tautomeric equilibrium is discussed based on the reactivity patterns given by the kinetic data over the reacting pairs. However, this analysis can be reinforced with the aid of computational and theoretical studies. Considering N-acetylcysteine as a reference nucleophile (Table 2 in this work) toward FDNB; the ratio with ethanolamine (PA) is 976 times and 17.5 times to piperazine (SAA). Then, N-acetylcysteine is more reactive than other nucleophiles of similar $pK_{a}$ values, but different in chemical nature. (Ormazábal-Toledo et al., 2013b). In summary, the studied substrates in reaction with these biothiols are highly reactive.

The Brönsted-type plots are shown in Supplementary Figures S25–S29 for each reaction studied. A Brönsted type-plot corresponds to a free energy relationship that correlates the logarithm of the nucleophilic rate coefficients and the $pK_{a}$ values of the nucleophiles from the Brönsted equation.

$$logk_{N}=\beta_{nuc}\;pK_{a}+log\;G\;,$$ (4)

where G is a constant that depends on the solvent and temperature and $\beta_{nuc}$ corresponds to the development of charge between reaction sites of the E^+^/Nu^−^ pair along to the potential energy surface (PES) (Brönsted, 1923; Brönsted and Pedersen, 1924). Therefore, $\beta_{nuc}$ provides information about the transition state (TS) structure related to the RDS on the reaction mechanism (Buncel et al., 1993). Brönsted-type plots for ClDNB showed $\beta_{nuc}=0.45\;\pm 0.07\;$ at 25°C and $\beta_{nuc}=0.46\;\pm 0.04$ at 37°C in aqueous media. On the other hand, the reported $\beta_{nuc}$ values in buffer phosphate were 0.42 ± 0.07 (at 25°C) and 0.48 ± 0.07 (at 37°C) where the contribution of buffer media and temperature show similar values. The Brönsted analysis for FDNB at 25°C reported a $\beta_{nuc}$ value of 0.43 ± 0.03. All the $\beta_{nuc}$ values are close, both substrates in agreement with the nucleophilic attack as RDS on a $S_{N}Ar_{stpw}$ mechanism ( $k_{1}\;$ in Figure 5), and the LG departure will be the fast step on the reaction route (Banjoko and Babatunde, 2004; Crampton et al., 2004; Terrier, 2013; Gallardo-Fuentes et al., 2014; Mortier, 2015). Although the RDS is the same for both substrates, –F is a better LG than–Cl with a general ratio close to 55 times (L-cysteine ethyl ester as reference). However, $k_{N}$ coefficients only reflect the first step of the reaction ( $k_{1}\;$ in Figure 5), because the LG departure takes place after the MC formation and the $k_{N}$ coefficient does not contain information about its nucleofugality (Nudelman et al., 1987; Alvaro et al., 2011; Ormazábal-Toledo et al., 2013b). Another possibility is to analyze the $\beta_{nuc}$ values associated with a $S_{N}Ar_{Conc}$ pathway. Recently, . Campodónico et al. (2020) published an interesting article based on Brönsted type-plot analysis for some S_N_Ar reactions where these might follow a concerted route (Um et al., 2014; Neumann et al., 2016; Neumann and Ritter, 2017; Kwan et al., 2018; Gallardo-Fuentes and Ormazábal-Toledo, 2019; Campodónico et al., 2020). Conversely, Kwan et al., (2018) suggested that chemical structures of substrates involved in the reaction play a key role in the reaction route in S_N_Ar reactions, specifically groups or atoms attached to the permanent groups (PG) and the nature of the LG in the substrate. An early study about nucleophilic substitution reactions was reported by Castro et al. (1999) based on concerted mechanisms for aminolysis of carboxylic esters derivatives, which have $\beta_{nuc}$ values in the range of 0.40–0.60. (Castro, 1999) The difference between nucleophilic substitution reactions and S_N_Ar reactions is the type of intermediate given by the nature of the reacting pair (Satterthwait and Jencks, 1974; Castro et al., 2002; Um et al., 2012). Therefore, in the context of our research, the substrates investigated are highly reactive, because they have two strong electron-withdrawing groups (-NO_2_ group) in *orto-* and *para-*position in the PG and good LG´s (-Cl and -F). Thus, the -NO_2_ groups promote the delocalization in the PG of the electrophile (substrates), which in conjunction with the LG departure might be activating the ipso carbon (electrophilic center) toward the nucleophilic attack. A comparative analysis of these electrophiles and others nucleophiles from our previous studies under the same experimental conditions have shown that: *i*) the reactivities of biothiols are determined by the chemical nature of the electrophile, *ii*) the reactions between atrazine toward biothiol series were reported as a borderline mechanism with slow rate coefficient values (Calfumán et al., 2017). On the other hand, FDNB reacting with secondary alicyclic (SA) amines and primary amines (PA) were reported as stepwise routes, where the nucleophilic attack was the RDS on the reaction mechanism.

**FIGURE 8:**
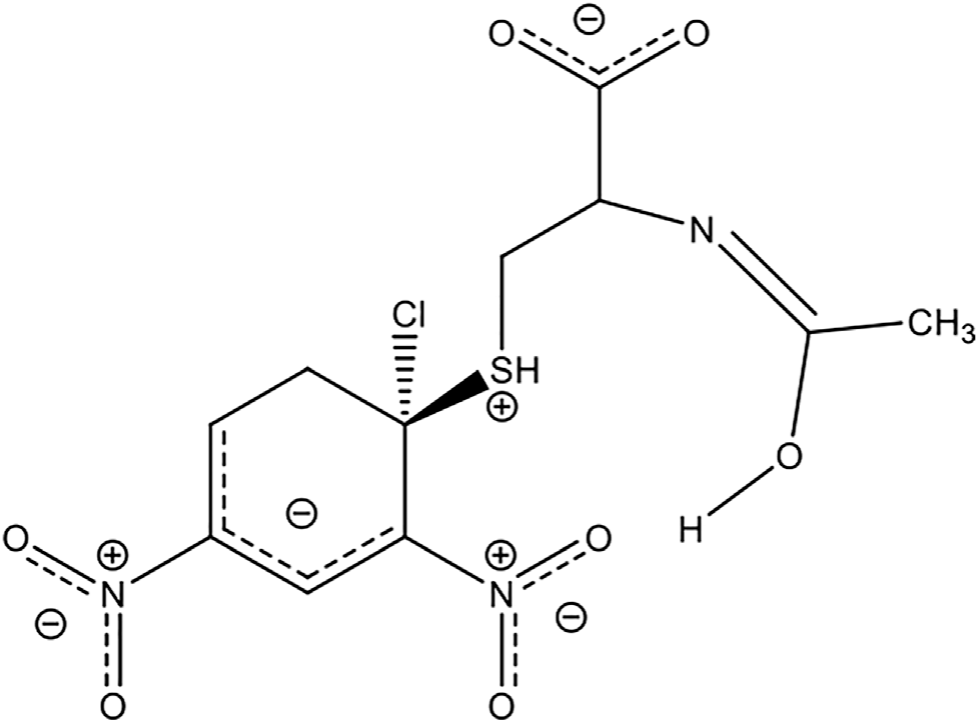
Representation of possible interaction between the substrate and N-acetylcysteine.

Then, $\beta_{nuc}$ values are contained in the range proposed for a concerted mechanism or stepwise route where the nucleophilic attack is the RDS. Then, considering the $\beta_{nuc}$ values and the Jacobsen trend (Kwan et al., 2018; Campodónico et al., 2020) in the S_N_Ar process of the mechanism for the reactions in this study are $S_{N}Ar_{Conc}$ or $S_{N}Ar_{stpw}$ borderline. Unfortunately, the biothiol series does not cover a substantial $pK_{a}$ range (6.5–9.5), but the Brönsted type-plots suggest that the TS structures associated with RDS are similar and the reactivities agree with their $pK_{a}$ values. In addition, the nature of the nucleophiles (anionic and protonated forms of the sulfhydryl group) mediated by the pH and the solvent effect are involved in the stabilization/destabilization of species along with the PES. Figure 8 shows a representation of the possible interaction between the substrate and N-acetylcysteine, wherein the intermediate species, the halogen (Cl- and F-) departure might be promoted by the hydrogen of water molecules from the reaction media and the hydrogen of the enol moiety from the tautomeric form, which may be stabilized by the ortho*-*nitro group of the PG of the substrate (Ormazábal-Toledo et al., 2013b; Bernasconi et al., 1976). In summary, the hydrogen bonding (HB) given by the reaction media and the reactivity patterns of the E^+^/Nu^−^ pairs can be promoted by the ability of the solvent to accept or donate HB and its polarity, which might explain the mechanistic trend, suggesting a concerted pathway for these studied reactions or close to a borderline stepwise route (Campodónico et al., 2020). A next contribution about the reaction mechanisms of these reacting pairs might be achieved integrating theoretical studies to our experimental analysis.

## Conclusion

We present a complete kinetic study based on S_N_Ar reactions. The Brönsted type-plots analysis of two known substrates with a series of biothiols suggest a concerted or borderline stepwise mechanism, where the amine–enol equilibrium established by N-acetylcysteine and glutathione toward these substrates appears as the main determining factor controlling the reactivity patterns toward a S_N_Ar reaction. This tautomeric form is associated with the chemical structure of these biothiols and hydrogen bonds from the aqueous media might be stabilizing the anionic form of the nucleophile and/or promoting the hydrogen departure from the -SH group and enhancing the nucleophilic strength toward the substrates. In addition, a complete product analysis suggests that the thiol group is the nucleophilic center discarding the amine group. Finally, it is relevant to highlight that some biological processes would be conditioned by reactivity patterns of the E^+^/Nu^−^ pairs involved in the reaction, which are demonstrated through their kinetic rates and reaction pathways.

## Data Availability

The original contributions presented in the study are included in the article/Supplementary Material; further inquiries can be directed to the corresponding author.
