# Supplementary material for: Kinetics and Reaction Mechanism of Biothiols Involved in SNAr Reactions: An Experimental Study
Source: Front Chem. 2022 Jun 8;10:854918. doi: 10.3389/fchem.2022.854918 (PMC9213796; doi:10.3389/fchem.2022.854918)

Supplementary Material

Kinetics and Reaction Mechanism of biothiols involved in S_NAr reactions. An Experimental Study.

Paola R. Campodónico[1]*, Jazmín Alarcón-Espósito[2] and Belén Olivares[1]

[1]Centro de Química Médica, Instituto de Ciencias e Innovación en Medicina, Facultad de Medicina, Clínica Alemana Universidad del Desarrollo, Santiago, Chile. [2]Departamento de Química Orgánica y Fisicoquímica, Facultad de Ciencias Químicas y Farmacéuticas, Universidad de Chile, Casilla 233, Santiago, Chile .

*** Correspondence:**
Corresponding Author
Paola Campodónico PhD.

pcampodonico@udd.cl

Figure S1. Plot of k_{obs} against free biothiol concentration $[Nu]_{free}$ for the reaction of 1-chloro-2,4-dinitrobenzene with L-Cysteine ethyl ester (CEE) in water solution at 25ºC±0.1ºC.

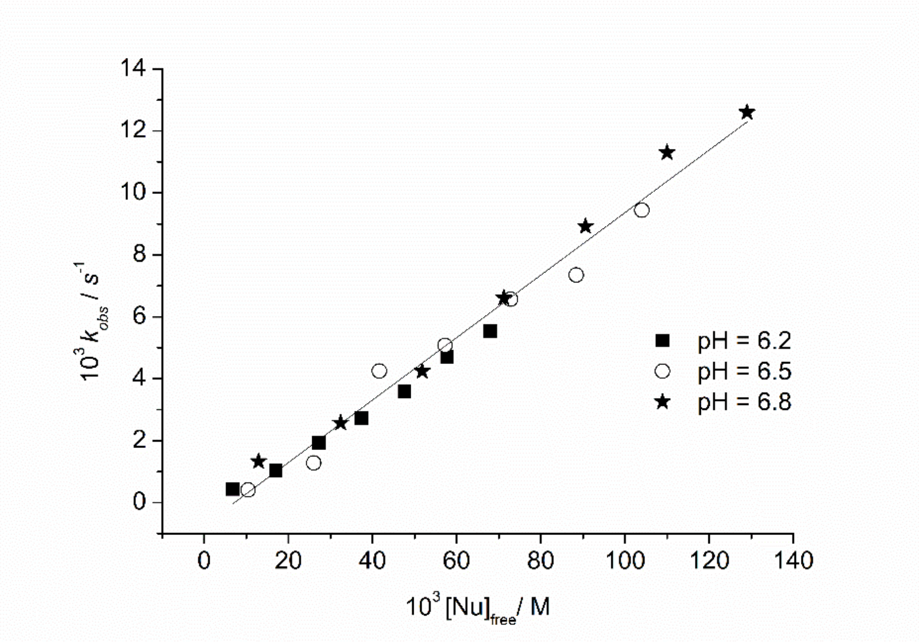

Figure S2. Plot of k_{obs} against free biothiol concentration $[Nu]_{free}$ for the reaction of 1-chloro-2,4-dinitrobenzene with L-Cysteine ethyl ester (CEE) in water solution at 37ºC±0.1ºC.

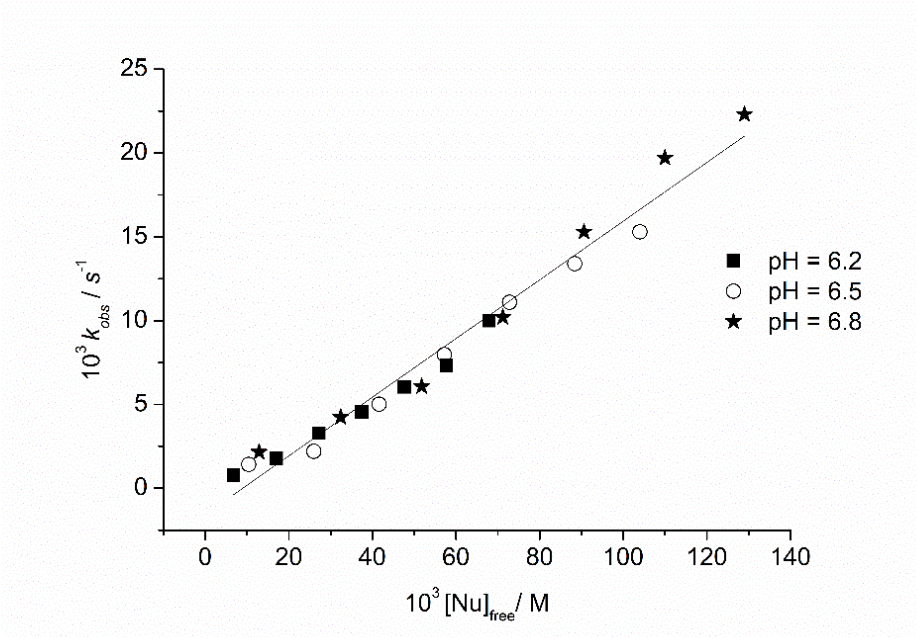

Figure S3. Plot of k_{obs} against free biothiol concentration $[Nu]_{free}$ for the reaction of 1-chloro-2,4-dinitrobenzene with L-Cysteine ethyl ester (CEE) in buffer phosphate media at 25ºC±0.1ºC.

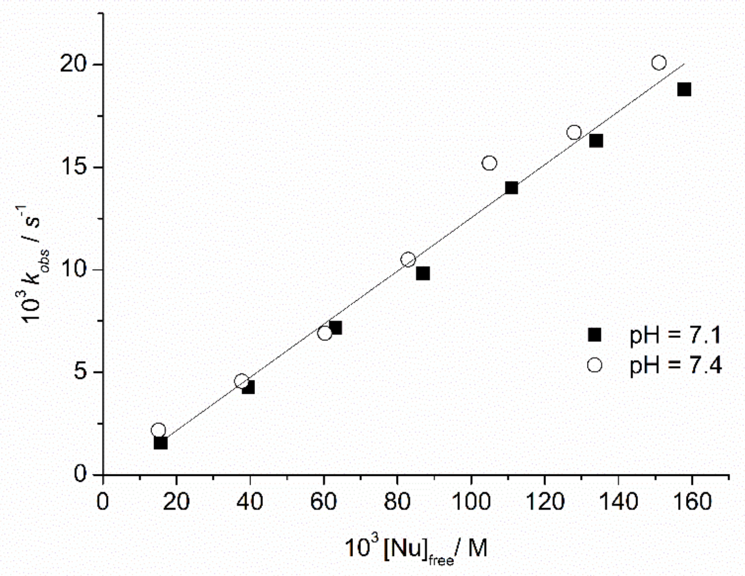

Figure S4. Plot of k_{obs} against free biothiol concentration $[Nu]_{free}$ for the reaction of 1-chloro-2,4-dinitrobenzene with L-Cysteine ethyl ester (CEE) in buffer phosphate media at 37ºC±0.1ºC.

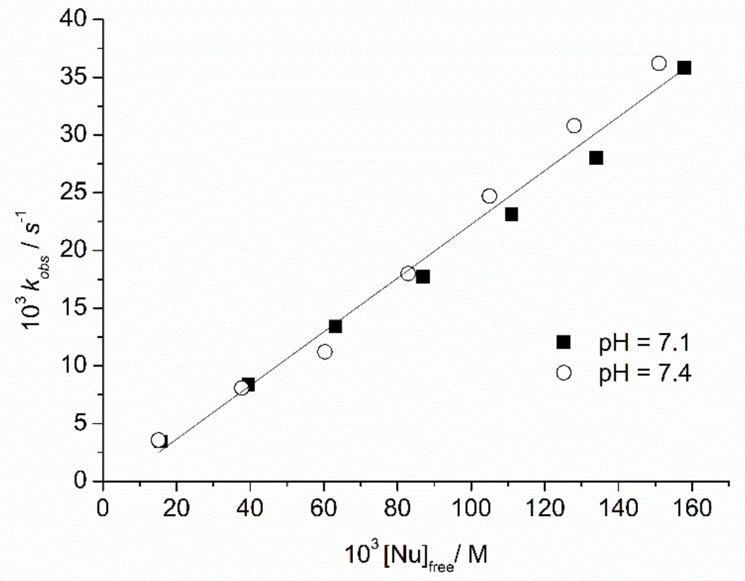

Figure S5. Plot of k_{obs} against free biothiol concentration $[Nu]_{free}$ for the reaction of 1-chloro-2,4-dinitrobenzene with Cysteine (Cyst) in water solution at 25ºC±0.1ºC.

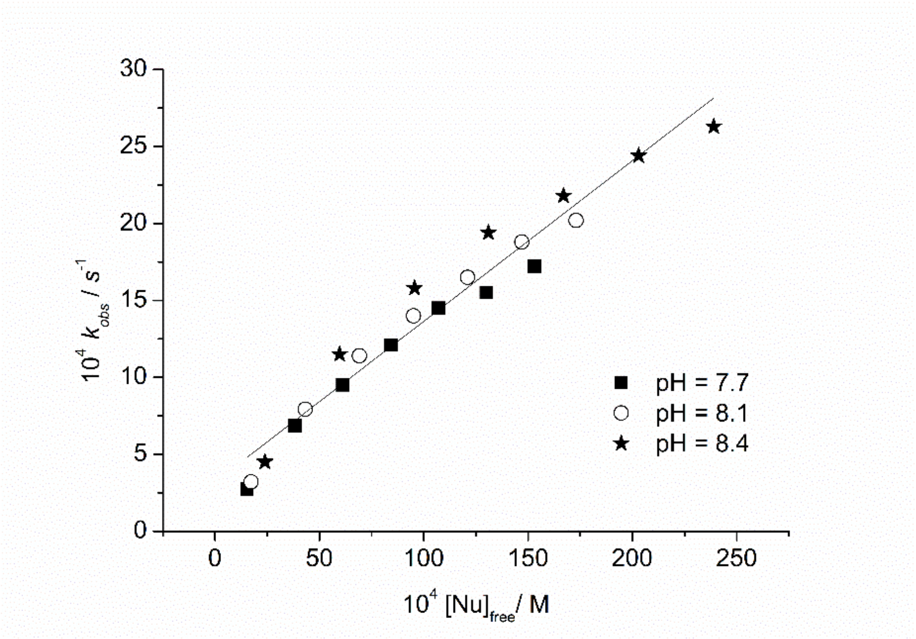

Figure S6. Plot of k_{obs} against free biothiol concentration $[Nu]_{free}$ for the reaction of 1-chloro-2,4-dinitrobenzene with Cysteine (Cyst) in water solution at 37ºC±0.1ºC.

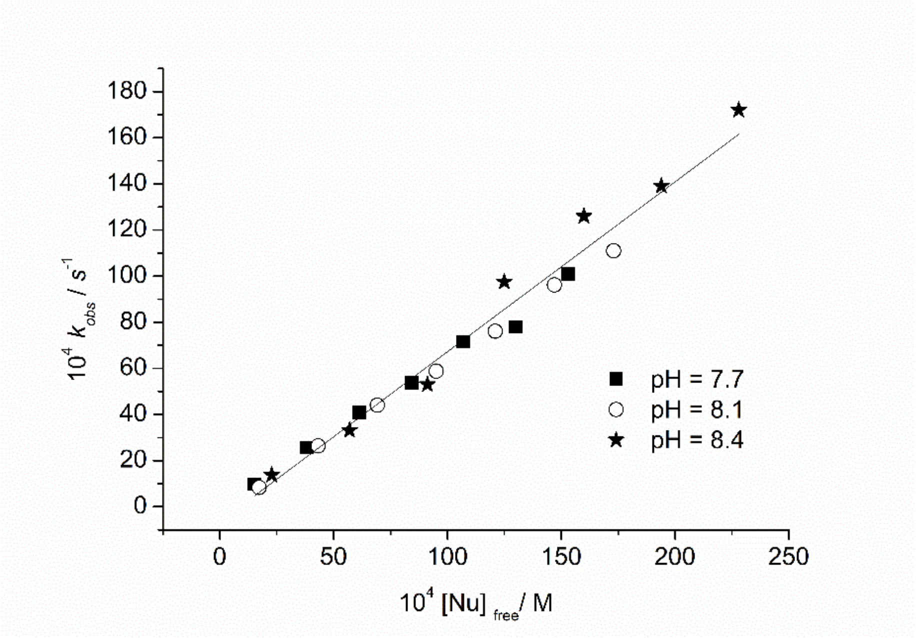

Figure S7. Plot of k_{obs} against free biothiol concentration $[Nu]_{free}$ for the reaction of 1-chloro-2,4-dinitrobenzene with Cysteine (Cyst) in buffer phosphate media at 25ºC±0.1ºC.

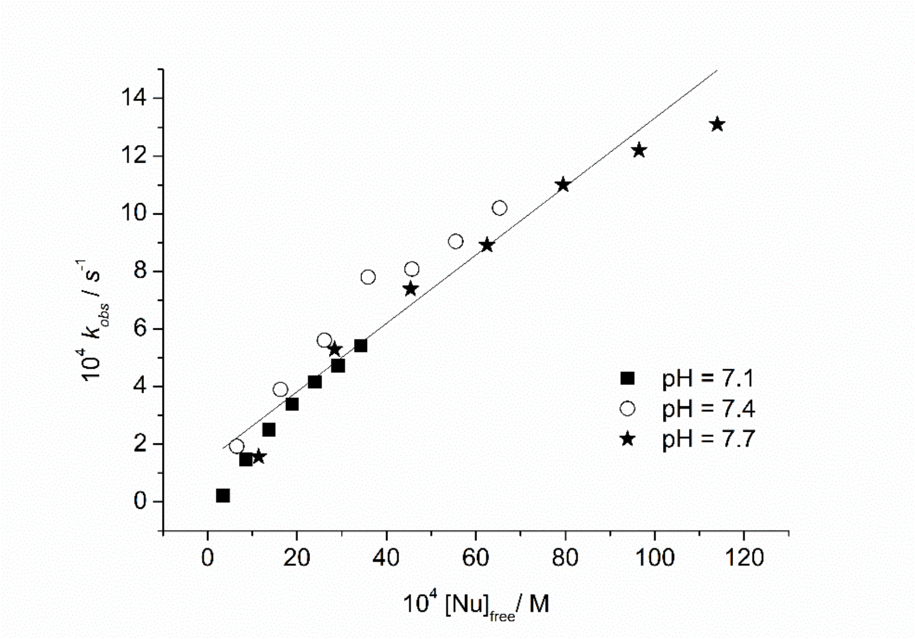

Figure S8. Plot of k_{obs} against free biothiol concentration $[Nu]_{free}$ for the reaction of 1-chloro-2,4-dinitrobenzene with Cysteine (Cyst) in buffer phosphate media at 37ºC±0.1ºC.

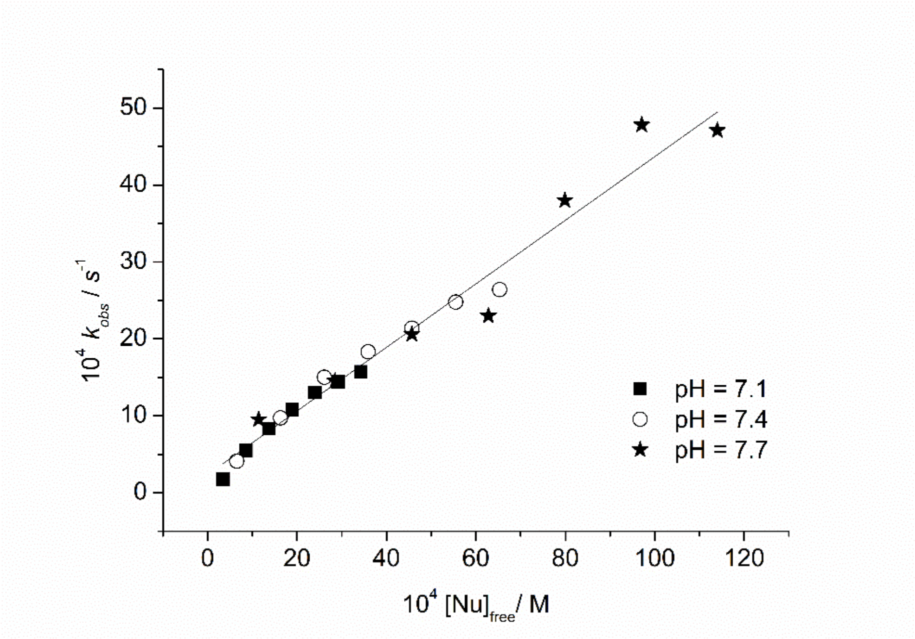

Figure S9. Plot of k_{obs} against free biothiol concentration $[Nu]_{free}$ for the reaction of 1-chloro-2,4-dinitrobenzene with DL-Homocysteine (HCyst) in water solution at 25ºC±0.1ºC.

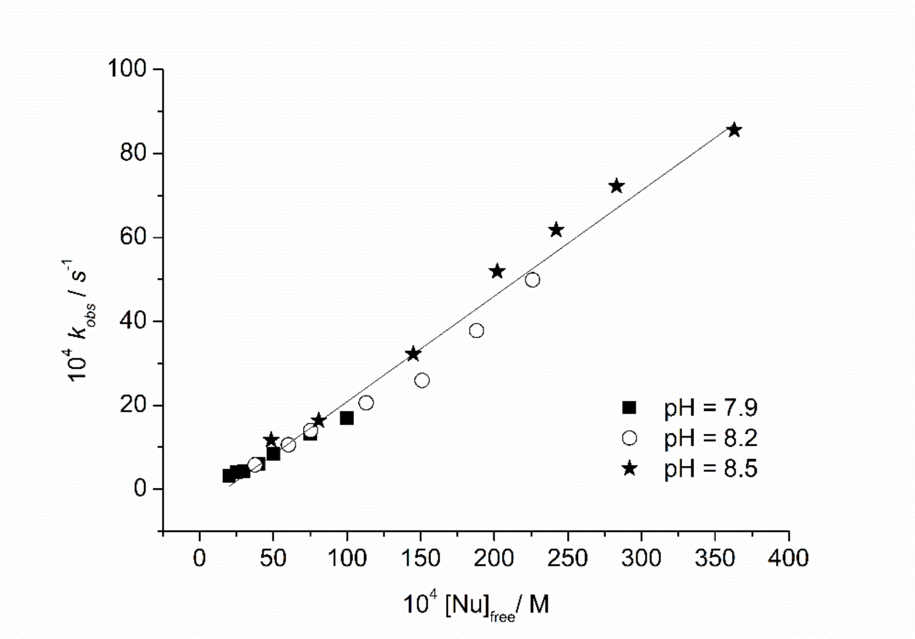

Figure S10. Plot of k_{obs} against free biothiol concentration $[Nu]_{free}$ for the reaction of 1-chloro-2,4-dinitrobenzene with DL-Homocysteine (HCyst) in water solution at 37ºC±0.1ºC.

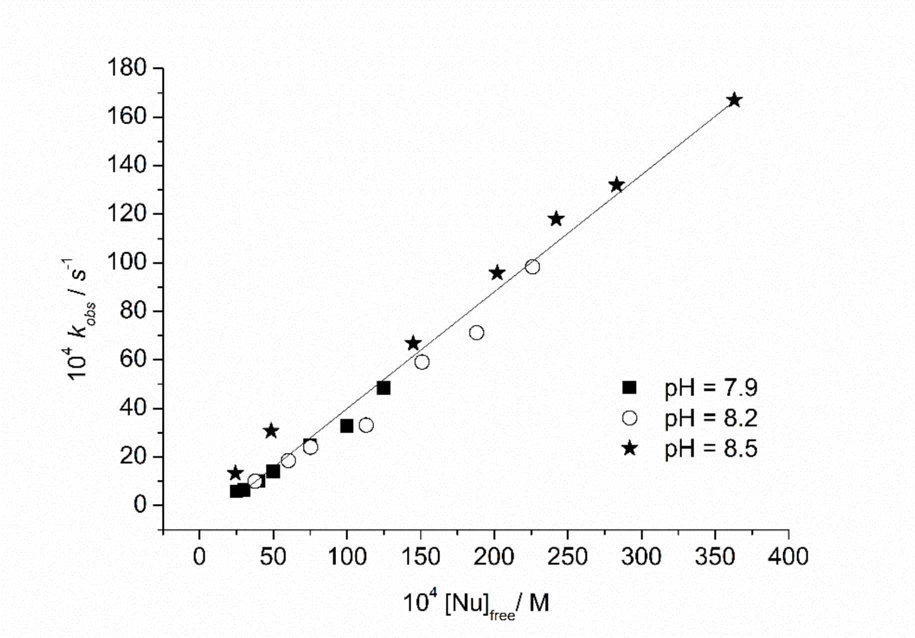

Figure S11. Plot of k_{obs} against free biothiol concentration $[Nu]_{free}$ for the reaction of 1-chloro-2,4-dinitrobenzene with DL-Homocysteine (HCyst) in buffer phosphate media at 25ºC±0.1ºC.

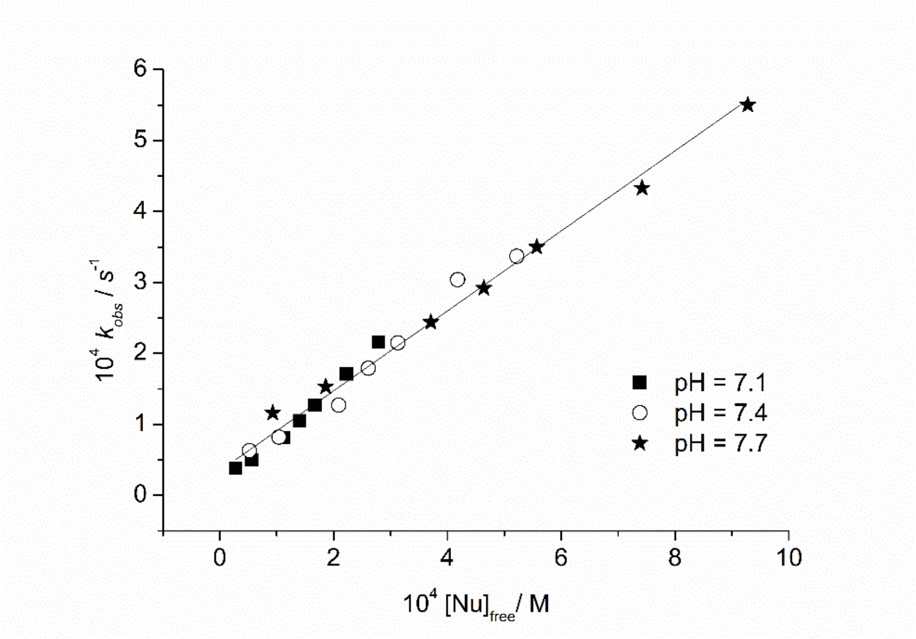

Figure S12. Plot of k_{obs} against free biothiol concentration $[Nu]_{free}$ for the reaction of 1-chloro-2,4-dinitrobenzene with DL-Homocysteine (HCyst) in buffer phosphate media at 37ºC±0.1ºC.

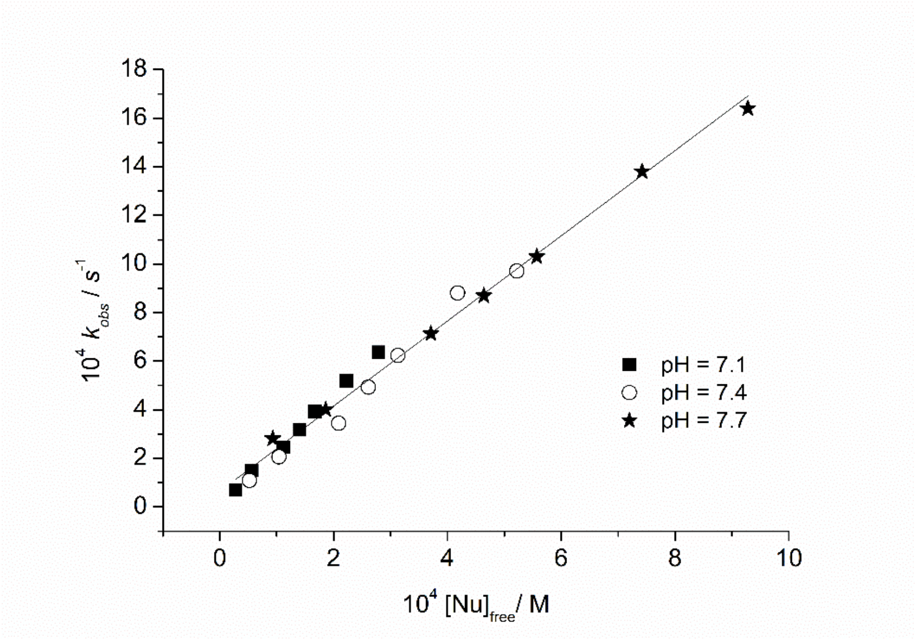

Figure S13. Plot of k_{obs} against free biothiol concentration $[Nu]_{free}$ for the reaction of 1-chloro-2,4-dinitrobenzene with Glutathione (GSH) in water solution at 25ºC±0.1ºC.

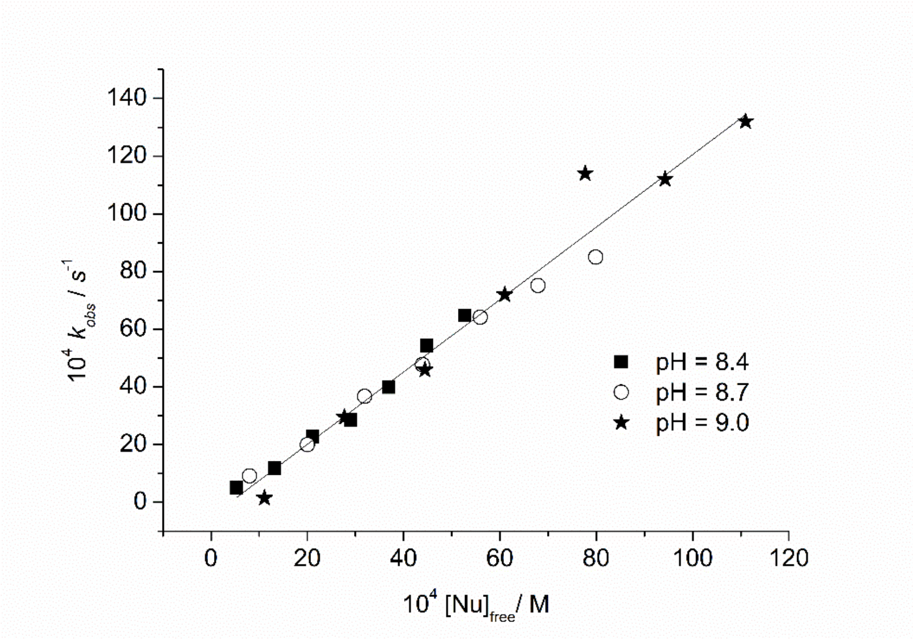

Figure S14. Plot of k_{obs} against free biothiol concentration $[Nu]_{free}$ for the reaction of 1-chloro-2,4-dinitrobenzene with Glutathione (GSH) in water solution at 37ºC±0.1ºC.

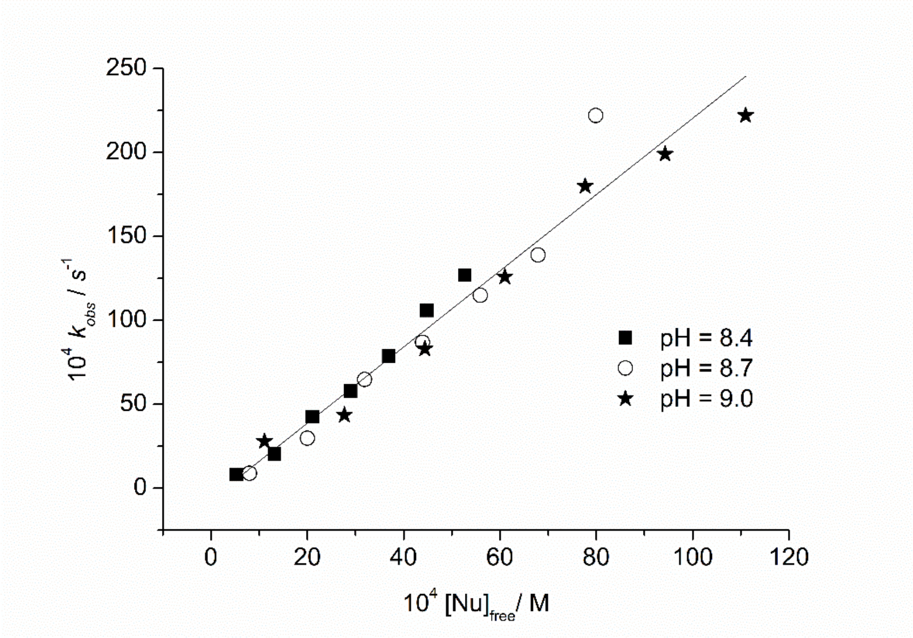

Figure S15. Plot of k_{obs} against free biothiol concentration $[Nu]_{free}$ for the reaction of 1-chloro-2,4-dinitrobenzene with Glutathione (GSH) in buffer phosphate media at 25ºC±0.1ºC.

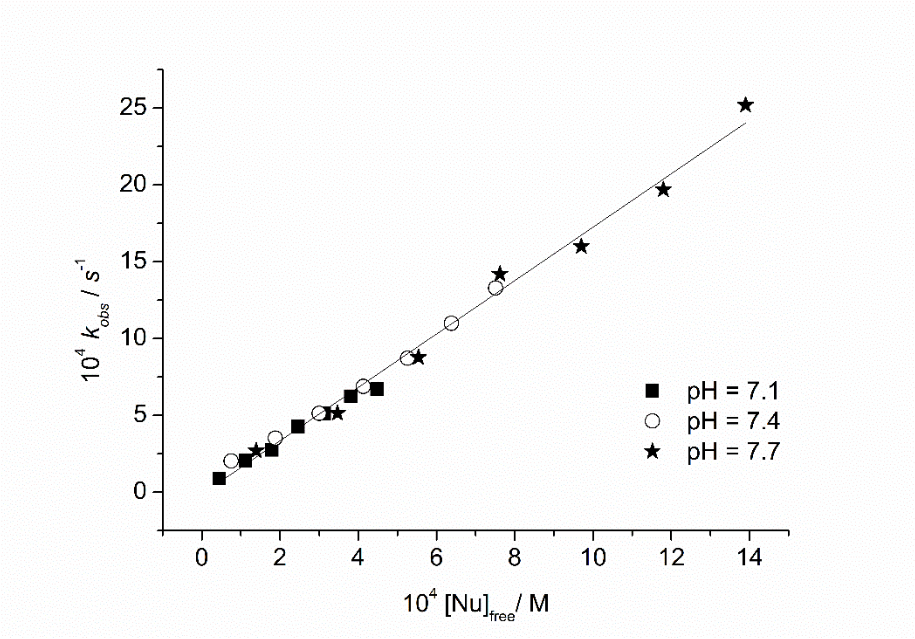

Figure S16. Plot of k_{obs} against free biothiol concentration $[Nu]_{free}$ for the reaction of 1-chloro-2,4-dinitrobenzene with Glutathione (GSH) in buffer phosphate media at 37ºC±0.1ºC.

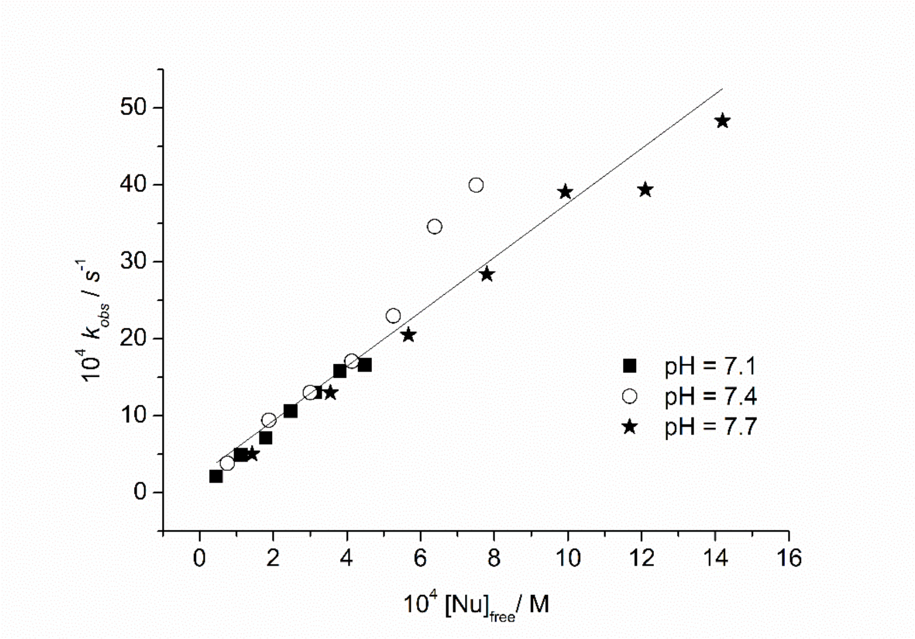

Figure S17. Plot of k_{obs} against free biothiol concentration $[Nu]_{free}$ for the reaction of 1-chloro-2,4-dinitrobenzene with N-acetylcysteine (NAC) in water solution at 25ºC±0.1ºC.

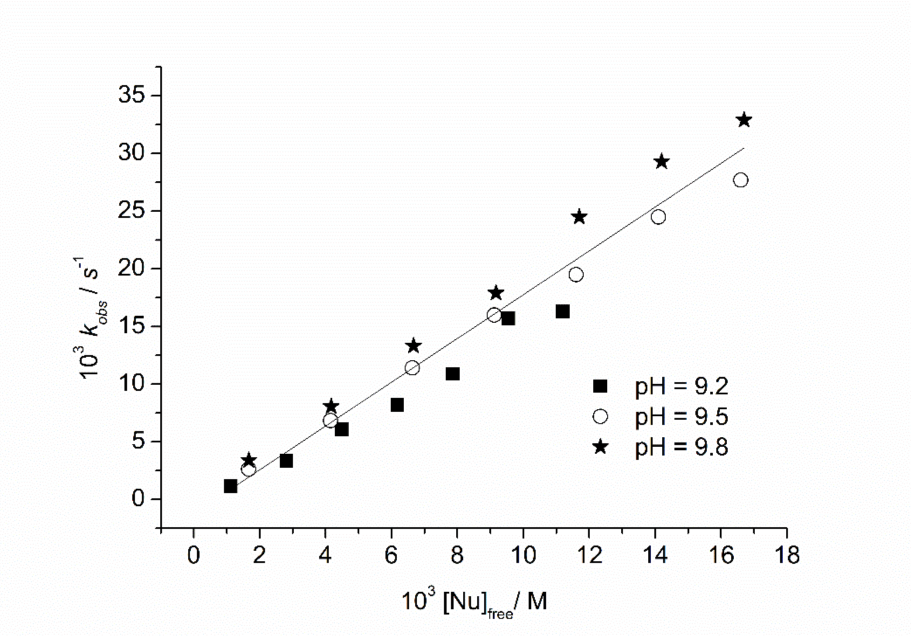

Figure S18. Plot of k_{obs} against free biothiol concentration $[Nu]_{free}$ for the reaction of 1-chloro-2,4-dinitrobenzene with N-acetylcysteine (NAC) in water solution at 37ºC±0.1ºC.

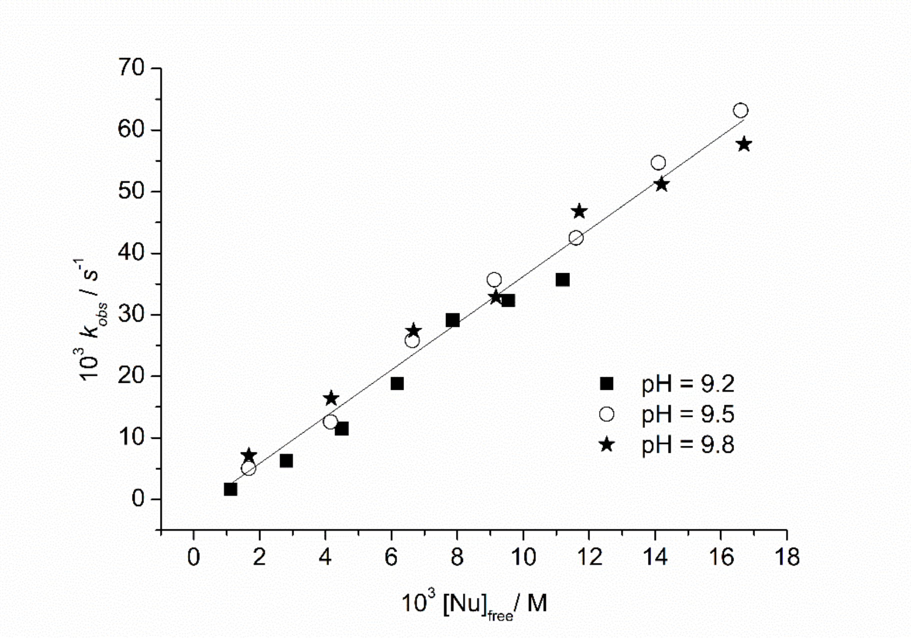

Figure S19. Plot of k_{obs} against free biothiol concentration $[Nu]_{free}$ for the reaction of 1-chloro-2,4-dinitrobenzene with N-acetylcysteine (NAC) in buffer phosphate media at 25ºC±0.1ºC.

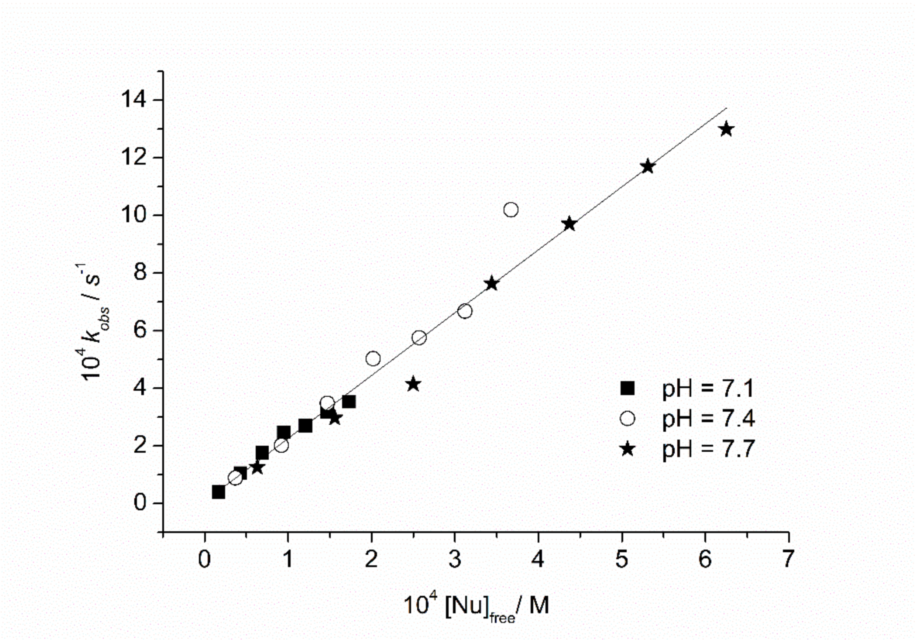

Figure S20. Plot of k_{obs} against free biothiol concentration $[Nu]_{free}$ for the reaction of 1-chloro-2,4-dinitrobenzene with N-acetylcysteine (NAC) in buffer phosphate media at 37ºC±0.1ºC.

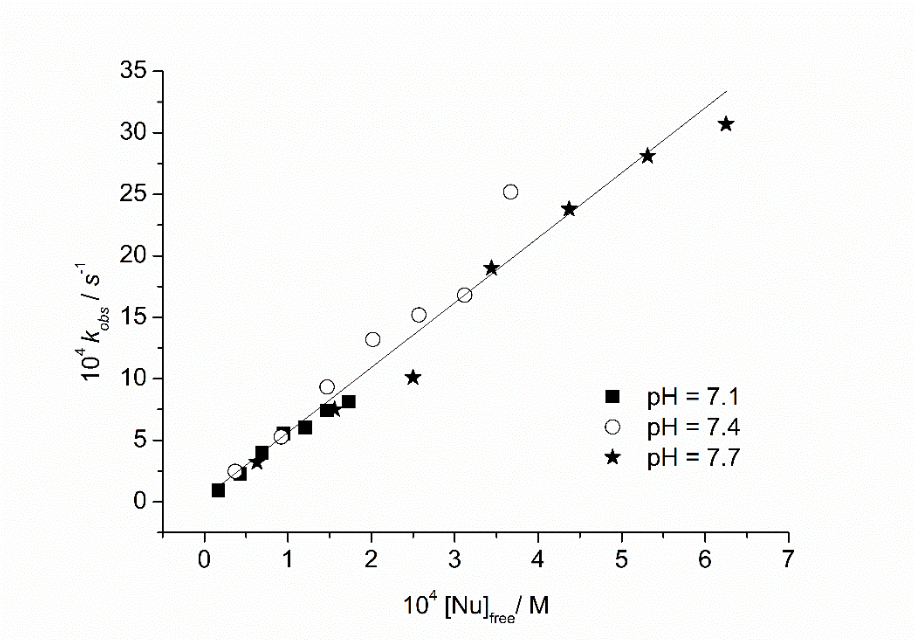

Figure S21. Plot of k_{obs} against free biothiol concentration $[Nu]_{free}$ for the reaction of 1-fluor-2,4-dinitrobenzene with L-Cysteine ethyl ester (CEE) in water solution at 25ºC±0.1ºC.

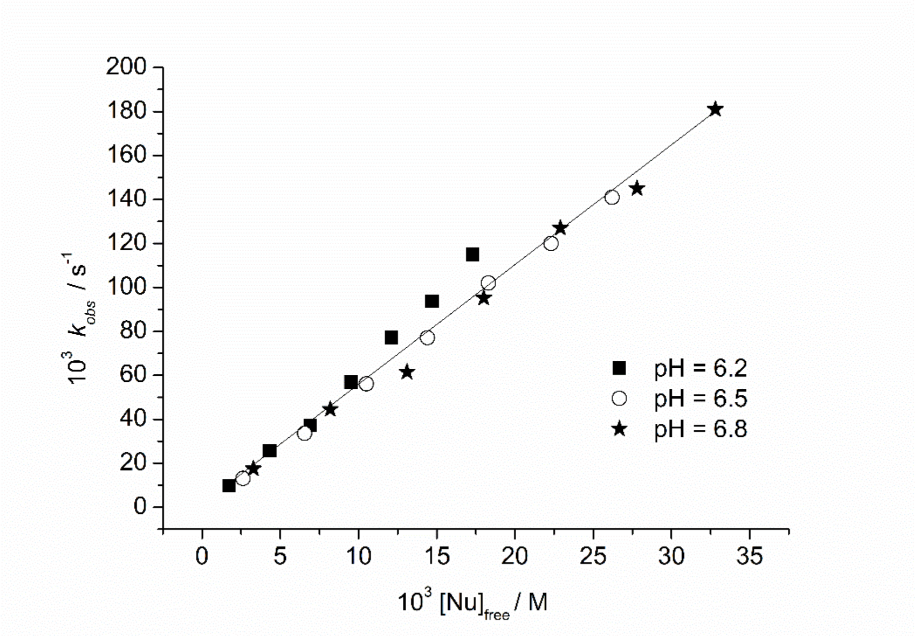

Figure S22. Plot of k_{obs} against free biothiol concentration $[Nu]_{free}$ for the reaction of 1-fluor-2,4-dinitrobenzene with N-acetylcysteine (NAC) in water solution at 25ºC±0.1ºC.

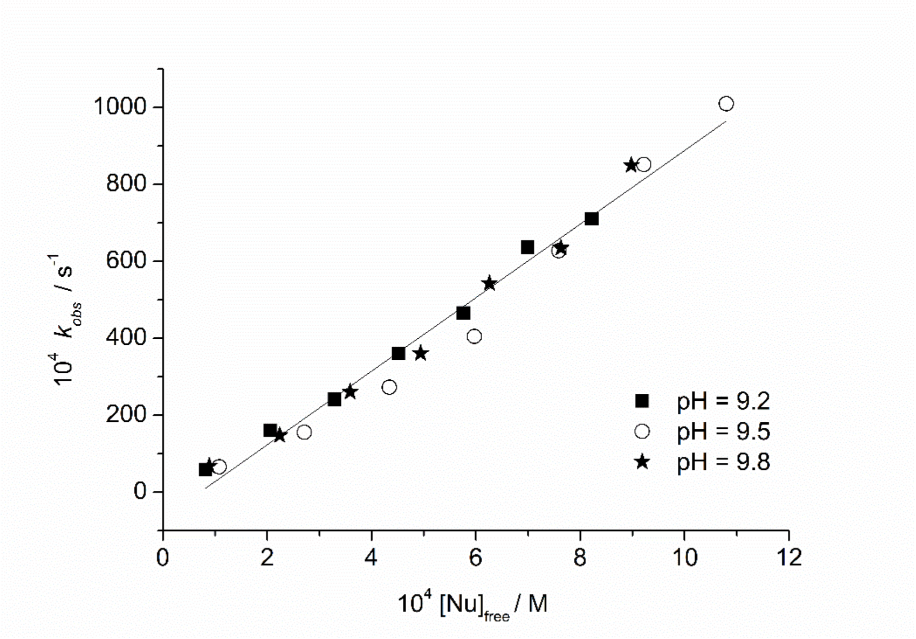

Figure S23. Plot of k_{obs} against free biothiol concentration $[Nu]_{free}$ for the reaction of 1-fluor-2,4-dinitrobenzene with Glutathione (GSH) in water solution at 25ºC±0.1ºC.

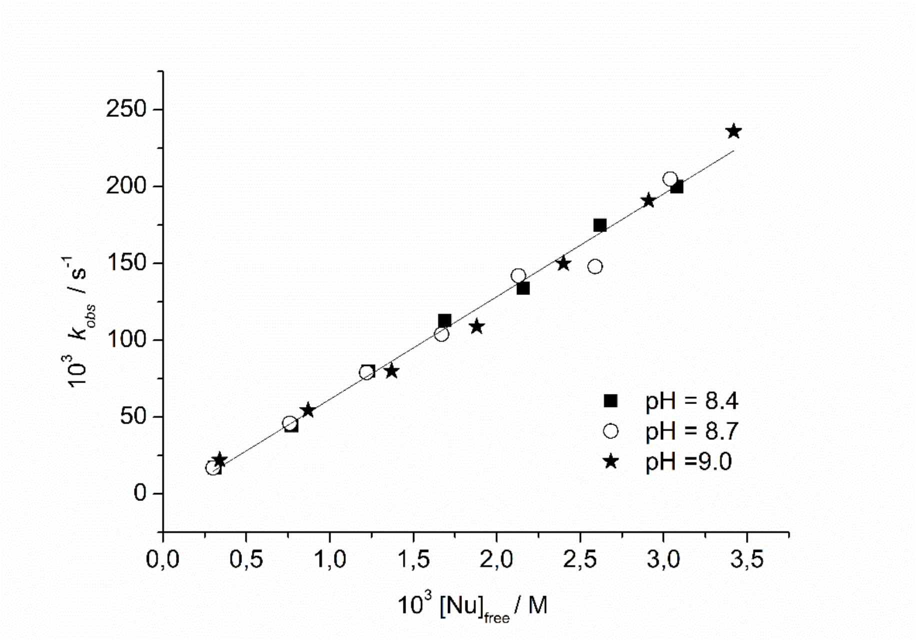

Figure S24. Plot of k_{obs} against free biothiol concentration $[Nu]_{free}$ for the reaction of 1-fluor-2,4-dinitrobenzene with Cysteine (Cyst) in water solution at 25ºC±0.1ºC.

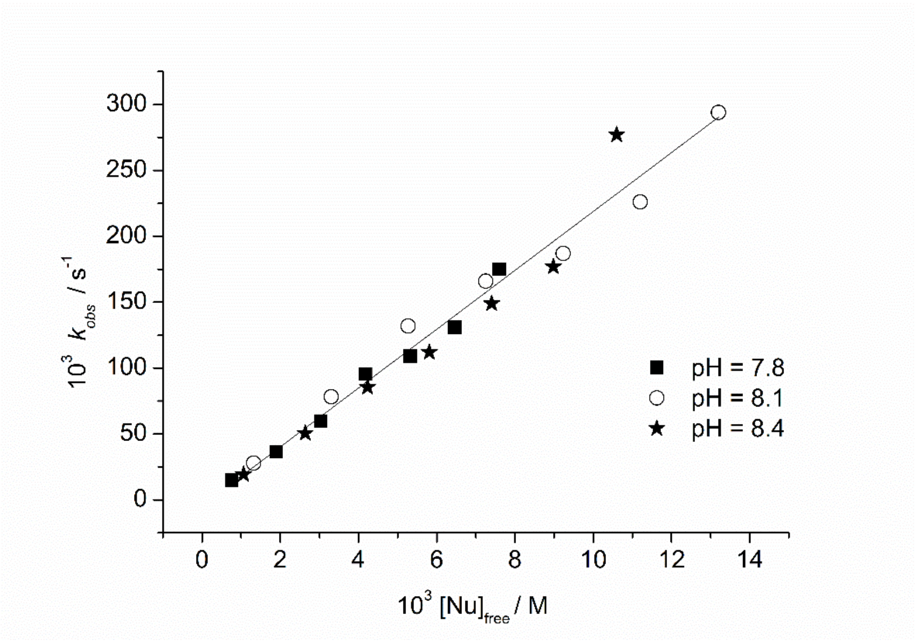

Table S1. Kinetic data for the reaction of 1-chloro-2,4-dinitrobenzene with L-Cysteine ethyl ester (CEE) in water solution at 25ºC±0.1ºC.

CEE pH=6.2		CEE pH=6.5		CEE pH=6.8	
$10^3[N]_{free}$ /M	$10^3k_{obs}/s^{-1}$	$10^3[N]_{free}$ /M	$10^3k_{obs}/s^{-1}$	$10^3[N]_{free}$ /M	$10^3k_{obs}/s^{-1}$
6.79	0.44	10.4	0.41	12.9	1.33
17.0	1.03	26.0	1.28	32.4	2.57
27.2	1.92	41.6	4.25	51.8	4.24
37.4	2.72	57.2	5.08	71.2	6.61
47.6	3.58	72.8	6.57	90.6	8.92
57.7	4.70	88.4	7.35	110	11.3
67.9	5.53	104	9.44	129	12.6

Table S2. Kinetic data for the reaction of 1-chloro-2,4-dinitrobenzene with L-Cysteine ethyl ester (CEE) in water solution at 37ºC±0.1ºC.

CEE pH=6.2		CEE pH=6.5		CEE pH=6.8	
$10^3[N]_{free}$ /M	$10^3k_{obs}/s^{-1}$	$10^3[N]_{free}$ /M	$10^3k_{obs}/s^{-1}$	$10^3[N]_{free}$ /M	$10^3k_{obs}/s^{-1}$
6.79	0.79	10.4	1.42	12.9	2.15
17.0	1.76	26.0	2.20	32.4	4.23
27.2	3.28	41.6	5.01	51.8	6.06
37.4	4.54	57.2	7.97	71.2	10.2
47.6	6.04	72.8	11.1	90.6	15.3
57.7	7.33	88.4	13.4	110	19.7
67.9	10.0	104	15.3	129	22.3

Table S3. Kinetic data for the reaction of 1-chloro-2,4-dinitrobenzene with L-Cysteine ethyl ester (CEE) in buffer phosphate media at 25ºC±0.1ºC.

CEE pH=7.1		CEE pH=7.4	
$10^3[N]_{free}$ /M	$10^3\ k_{obs}/s^{-1}$	$10^3\ [N]_{free}$ /M	$10^3\ k_{obs}/s^{-1}$
15.8	1.55	15.1	2.17
39.5	4.28	37.7	4.56
63.2	7.16	60.3	6.90
86.9	9.82	82.9	10.5
111	14.0	105	15.2
134	16.3	128	16.7
158	18.8	151	20.1

Table S4. Kinetic data for the reaction of 1-chloro-2,4-dinitrobenzene with L-Cysteine ethyl ester (CEE) in buffer phosphate media at 37ºC±0.1ºC.

CEE pH=7.1		CEE pH=7.4	
$10^3[N]_{free}$ /M	$10^3\ k_{obs}/s^{-1}$	$10^3\ [N]_{free}$ /M	$10^3\ k_{obs}/s^{-1}$
15.8	3.43	15.1	3.57
39.5	8.36	37.7	8.08
63.2	13.4	60.3	11.2
86.9	17.7	82.9	18.0
111	23.1	105	24.7
134	28.0	128	30.8
158	35.8	151	36.2

Table S5. Kinetic data for the reaction of 1-chloro-2,4-dinitrobenzene with Cysteine (Cyst) in water solution at 25ºC±0.1ºC.

Cyst pH=7.7		Cyst pH=8.1		Cyst pH=8.4	
$10^4[N]_{free}$ /M	$10^4k_{obs}/s^{-1}$	$10^4[N]_{free}$ /M	$10^4k_{obs}/s^{-1}$	$10^4[N]_{free}$ /M	$10^4k_{obs}/s^{-1}$
15.3	2.74	17.3	3.21	23.9	4.50
38.3	6.85	43.2	7.93	59.7	11.5
61.2	9.51	69.2	11.4	95.6	15.8
84.2	12.1	95.1	14.0	131	19.4
107	14.5	121	16.5	167	21.8
130	15.5	147	18.8	203	24.4
153	17.2	173	20.2	239	26.3

Table S6. Kinetic data for the reaction of 1-chloro-2,4-dinitrobenzene with Cysteine (Cyst) in water solution at 37ºC±0.1ºC.

Cyst pH=7.7		Cyst pH=8.1		Cyst pH=8.4	
$10^4[N]_{free}$ /M	$10^4k_{obs}/s^{-1}$	$10^4[N]_{free}$ /M	$10^4k_{obs}/s^{-1}$	$10^4[N]_{free}$ /M	$10^4k_{obs}/s^{-1}$
15.3	9.78	17.3	8.38	22.8	13.7
38.3	25.6	43.2	26.4	57.0	33.1
61.3	40.8	69.2	44.0	91.2	53.0
84.3	53.7	95.1	58.8	125	97.5
107	71.5	121	76.1	160	126
130	78.0	147	96.2	194	139
153	101	173	111	228	172

Table S7. Kinetic data for the reaction of 1-chloro-2,4-dinitrobenzene with Cysteine (Cyst) in buffer phosphate media at 25ºC±0.1ºC.

Cyst pH=7.1		Cyst pH=7.4		Cyst pH=7.7	
$10^4[N]_{free}$ /M	$10^4k_{obs}/s^{-1}$	$10^4[N]_{free}$ /M	$10^4k_{obs}/s^{-1}$	$10^4[N]_{free}$ /M	$10^4k_{obs}/s^{-1}$
3.43	0.22	6.53	1.93	11.4	1.57
8.58	1.47	16.3	3.90	28.4	5.29
13.7	2.50	26.1	5.61	45.4	7.39
18.9	3.39	35.9	7.80	62.5	8.91
24.0	4.16	45.7	8.08	79.5	11.0
29.2	4.72	55.5	9.04	96.5	12.2
34.3	5.41	65.3	10.2	114	13.1

Table S8. Kinetic data for the reaction of 1-chloro-2,4-dinitrobenzene with Cysteine (Cyst) in buffer phosphate media at 37ºC±0.1ºC.

Cyst pH=7.1		Cyst pH=7.4		Cyst pH=7.7	
$10^4[N]_{free}$ /M	$10^4k_{obs}/s^{-1}$	$10^4[N]_{free}$ /M	$10^4k_{obs}/s^{-1}$	$10^4[N]_{free}$ /M	$10^4k_{obs}/s^{-1}$
3.43	1.72	6.53	4.10	11.4	9.51
8.58	5.50	16.3	9.72	28.5	14.5
13.7	8.31	26.1	15.0	45.7	20.6
18.9	10.8	35.9	18.3	62.8	23.0
24.0	13.0	45.7	21.4	79.9	38.0
29.2	14.4	55.5	24.8	97.1	47.8
34.3	15.7	65.3	26.4	114	47.1

Table S9. Kinetic data for the reaction of 1-chloro-2,4-dinitrobenzene with DL-Homocysteine (HCys) in water solution at 25ºC±0.1ºC.

HCys pH=7.9		HCys pH=8.2		HCys pH=8.5	
$10^4[N]_{free}$ /M	$10^4k_{obs}/s^{-1}$	$10^4[N]_{free}$ /M	$10^4k_{obs}/s^{-1}$	$10^4[N]_{free}$ /M	$10^4k_{obs}/s^{-1}$
20.0	3.21	37.6	5.82	48.5	11.7
25.0	4.01	60.2	10.6	80.8	16.4
30.0	4.27	75.3	14.0	145	32.2
40.0	6.02	113	20.6	202	51.9
50.0	8.43	151	25.9	242	61.8
74.9	13.1	188	37.8	283	72.2
99.9	17.0	226	49.9	363	85.6

Table S10. Kinetic data for the reaction of 1-chloro-2,4-dinitrobenzene with DL-Homocysteine (HCys) in water solution at 37ºC±0.1ºC.

HCys pH=7.9		HCys pH=8.2		HCys pH=8.5	
$10^4[N]_{free}$ /M	$10^4k_{obs}/s^{-1}$	$10^4[N]_{free}$ /M	$10^4k_{obs}/s^{-1}$	$10^4[N]_{free}$ /M	$10^4k_{obs}/s^{-1}$
25.0	5.78	37.6	10.0	24.2	13.2
30.0	6.41	60.2	18.5	48.5	30.7
40.0	10.1	75.3	24.0	145	66.7
50.0	14.0	113	33.1	202	95.8
74.9	24.9	151	59.1	242	118
99.9	32.8	188	71.2	283	132
125	48,4	226	98,3	363	167

Table S11. Kinetic data for the reaction of 1-chloro-2,4-dinitrobenzene with DL-Homocysteine (HCys) in buffer phosphate media at 25ºC±0.1ºC.

HCys pH=7.1		HCys pH=7.4		HCys pH=7.7	
$10^4[N]_{free}$ /M	$10^4k_{obs}/s^{-1}$	$10^4[N]_{free}$ /M	$10^4k_{obs}/s^{-1}$	$10^4[N]_{free}$ /M	$10^4k_{obs}/s^{-1}$
0.28	0.38	0.52	0.63	0.93	1.16
0.56	0.50	1.04	0.82	1.86	1.53
1.12	0.81	2.09	1.27	3.71	2.44
1.40	1.05	2.61	1.79	4.64	2.92
1.67	1.27	3.13	2.15	5.57	3.50
2.23	1.71	4.18	3.04	7.42	4.33
2.79	2.16	5.22	3.37	9.28	5.50

Table S12. Kinetic data for the reaction of 1-chloro-2,4-dinitrobenzene with DL-Homocysteine (HCys) in buffer phosphate media at 37ºC±0.1ºC.

HCys pH=7.1		HCys pH=7.4		HCys pH=7.7	
$10^4[N]_{free}$ /M	$10^4k_{obs}/s^{-1}$	$10^4[N]_{free}$ /M	$10^4k_{obs}/s^{-1}$	$10^4[N]_{free}$ /M	$10^4k_{obs}/s^{-1}$
0.28	0.69	0.52	1.09	0.93	2.81
0.56	1.50	1.04	2.06	1.86	4.01
1.12	2.45	2.09	3.44	3.71	7.13
1.40	3.17	2.61	4.93	4.64	8.69
1.67	3.92	3.13	6.23	5.57	10.3
2.23	5.18	4.18	8.81	7.42	13.8
2.79	6.36	5.22	9.71	9.28	16.4

Table S13. Kinetic data for the reaction of 1-chloro-2,4-dinitrobenzene with Glutathione (GSH) in water solution at 25ºC$\pm$0.1ºC.

GSH pH=8.4		GSH pH=8.7		GSH pH=9.0	
$10^4[N]_{free}$ /M	$10^4k_{obs}/s^{-1}$	$10^4[N]_{free}$ /M	$10^4k_{obs}/s^{-1}$	$10^4[N]_{free}$ /M	$10^4k_{obs}/s^{-1}$
5.27	5.07	7.99	9.18	11.1	1.51
13.2	11.9	20.0	20.0	27.7	29.5
21.1	22.8	31.9	36.7	44.4	45.9
29.0	28.5	43.9	47.7	61.0	72.0
36.9	39.9	55.9	64.2	77.7	114
44.8	54.3	67.9	75.2	94.3	112
52.7	64.8	79.9	85.0	111	132

Table S14. Kinetic data for the reaction of 1-chloro-2,4-dinitrobenzene with Glutathione (GSH) in water solution at 37ºC±0.1ºC.

GSH pH=8.4		GSH pH=8.7		GSH pH=9.0	
$10^4[N]_{free}$ /M	$10^4k_{obs}/s^{-1}$	$10^4[N]_{free}$ /M	$10^4k_{obs}/s^{-1}$	$10^4[N]_{free}$ /M	$10^4k_{obs}/s^{-1}$
5.27	8.08	7.99	8.94	11.1	27.9
13.2	20.4	20.0	29.8	27.7	43.6
21.1	42.6	31.9	64.8	44.4	83.0
29.0	57.9	43.9	87.0	61.0	126
36.9	78.7	55.9	115	77.7	180
44.8	106	67.9	139	94.3	199
52.7	127	79.9	222	111	222

Table S15. Kinetic data for the reaction of 1-chloro-2,4-dinitrobenzene with Glutathione (GSH) in buffer phosphate media at 25ºC±0.1ºC.

GSH pH=7.1		GSH pH=7.4		GSH pH=7.7	
$10^4[N]_{free}$ /M	$10^4k_{obs}/s^{-1}$	$10^4[N]_{free}$ /M	$10^4k_{obs}/s^{-1}$	$10^4[N]_{free}$ /M	$10^4k_{obs}/s^{-1}$
0.45	0.90	0.75	2.04	1.39	2.70
1.12	2.05	1.88	3.52	3.47	5.14
1.79	2.75	3.00	5.13	5.54	8.77
2.46	4.27	4.13	6.89	7.62	14.2
3.13	5.13	5.26	8.72	9.70	16.0
3.81	6.24	6.38	11.0	11.8	19.7
4.48	6.70	7.51	13.3	13.9	25.2

Table S16. Kinetic data for the reaction of 1-chloro-2,4-dinitrobenzene with Glutathione (GSH) in buffer phosphate media at 37ºC±0.1ºC.

GSH pH=7.1		GSH pH=7.4		GSH pH=7.7	
$10^4[N]_{free}$ /M	$10^4k_{obs}/s^{-1}$	$10^4[N]_{free}$ /M	$10^4k_{obs}/s^{-1}$	$10^4[N]_{free}$ /M	$10^4k_{obs}/s^{-1}$
0.45	2.12	0.75	3.82	1.42	5.03
1.12	4.92	1.88	9.43	3.55	13.0
1.79	7.11	3.00	13.0	5.67	20.5
2.47	10.6	4.13	17.1	7.80	28.4
3.14	13.0	5.26	23.0	9.93	39.1
3.81	15.8	6.38	34.6	12.1	39.4
4.48	16.6	7.51	40.0	14.2	48.3

Table S17. Kinetic data for the reaction of 1-chloro-2,4-dinitrobenzene with N-Acetyl-Cysteine (NAC) in water solution at 25ºC±0.1ºC.

NAC pH=9.2		NAC pH=9.5		NAC pH=9.8	
10^3[N]$_{free}$ /M	10^3k$_{obs}$/s^{-1}	10^3[N]$_{free}$ /M	10^3k$_{obs}$/s^{-1}	10^3[N]$_{free}$ /M	10^3k$_{obs}$/s^{-1}
1.12	1.15	1.66	2.63	1.67	3.39
2.81	3.35	4.15	6.84	4.17	8.06
4.49	6.07	6.63	11.4	6.67	13.3
6.18	8.20	9.12	16.0	9.17	17.9
7.86	10.9	11.6	19.5	11.7	24.5
9.55	15.7	14.1	24.5	14.2	29.3
11.2	16.3	16.6	27.7	16.7	32.9

Table S18. Kinetic data for the reaction of 1-chloro-2,4-dinitrobenzene with N-Acetyl-Cysteine (NAC) in water solution at 37ºC±0.1ºC.

NAC pH=9.2		NAC pH=9.5		NAC pH=9.8	
$10^3[N]_{free}$ /M	$10^3k_{obs}/s^{-1}$	$10^3[N]_{free}$ /M	$10^3k_{obs}/s^{-1}$	$10^3[N]_{free}$ /M	$10^3k_{obs}/s^{-1}$
1.12	1.61	1.66	5.06	1.67	7.11
2.81	6.27	4.15	12.6	4.17	16.4
4.49	11.5	6.63	25.8	6.67	27.4
6.18	18.8	9.12	35.7	9.17	32.9
7.86	29.1	11.6	42.5	11.7	46.8
9.55	32.3	14.1	54.7	14.2	51.2
11.2	35.7	16.6	63.2	16.7	57.7

Table S19. Kinetic data for the reaction of 1-chloro-2,4-dinitrobenzene with N-acetyl-Cysteine (NAC) in buffer phosphate media at 25ºC$\pm$0.1ºC.

NAC pH=7.1		NAC pH=7.4		NAC pH=7.7	
$10^4[N]_{free}$ /M	$10^4k_{obs}/s^{-1}$	$10^4[N]_{free}$ /M	$10^4k_{obs}/s^{-1}$	$10^4[N]_{free}$ /M	$10^4k_{obs}/s^{-1}$
0.17	0.40	0.37	0.89	0.63	1.26
0.43	1.06	0.92	2.02	1.56	2.97
0.69	1.76	1.47	3.48	2.50	4.15
0.95	2.47	2.02	5.03	3.44	7.64
1.21	2.70	2.57	5.76	4.37	9.71
1.47	3.17	3.12	6.68	5.31	11.7
1.73	3.53	3.67	10.2	6.25	13.0

Table S20. Kinetic data for the reaction of 1-chloro-2,4-dinitrobenzene with N-acetyl-Cysteine (NAC) in buffer phosphate media at 37ºC±0.1ºC.

NAC pH=7.1		NAC pH=7.4		NAC pH=7.7	
10^4[N]$_{free}$ /M	10^4k$_{obs}$/s^{-1}	10^4[N]$_{free}$ /M	10^4k$_{obs}$/s^{-1}	10^4[N]$_{free}$ /M	10^4k$_{obs}$/s^{-1}
0.17	0.91	0.37	2.48	0.63	3.21
0.43	2.26	0.92	5.27	1.56	7.51
0.69	3.99	1.47	9.33	2.50	10.1
0.95	5.54	2.02	13.2	3.44	19.0
1.21	6.04	2.57	15.2	4.37	23.8
1.47	7.43	3.12	16.8	5.31	28.1
1.73	8.14	3.67	25.2	6.25	30.7

Table S21. Kinetic data for the reaction of 1-fluor-2,4-dinitrobenzene with L-Cysteine ethyl ester (CEE) in water solution at 25ºC±0.1ºC.

CEE pH=6.2		CEE pH=6.5		CEE pH=6.8	
$10^3[N]_{free}$ /M	$10^3k_{obs}/s^{-1}$	$10^3[N]_{free}$ /M	$10^3k_{obs}/s^{-1}$	$10^3[N]_{free}$ /M	$10^3k_{obs}/s^{-1}$
1.73	9.83	2.62	13.1	3.28	17.5
4.33	25.7	6.55	33.6	8.19	44.5
6.93	37.2	10.5	56.1	13.1	61.5
9.53	56.9	14.4	77.1	18.0	95.2
12.1	77.2	18.3	102	22.9	127
14.7	93.8	22.3	120	27.8	145
17.3	115	26.2	141	32.8	181

Table S22. Kinetic data for the reaction of 1-fluor-2,4-dinitrobenzene with N-Acetyl-Cysteine (NAC) in water solution at 25ºC±0.1ºC.

NAC pH=9.2		NAC pH=9.5		NAC pH=9.8	
$10^4[N]_{free}$ /M	$10^4k_{obs}/s^{-1}$	$10^4[N]_{free}$ /M	$10^4k_{obs}/s^{-1}$	$10^4[N]_{free}$ /M	$10^4k_{obs}/s^{-1}$
0.82	59	1.08	66.2	0.89	68.1
2.06	161	2.71	156	2.24	148
3.29	241	4.34	273	3.59	261
4.52	361	5.97	405	4.94	361
5.76	466	7.59	628	6.26	543
6.99	637	9.22	852	7.63	635
8.22	711	10.8	1010	8.98	850

Table S23. Kinetic data for the reaction of 1-fluor-2,4-dinitrobenzene with Glutathione (GSH) in water solution at 25ºC±0.1ºC.

GSH pH=8.4		GSH pH=8.7		GSH pH=9.0	
$10^3[N]_{free}$ /M	$10^3k_{obs}/s^{-1}$	$10^3[N]_{free}$ /M	$10^3k_{obs}/s^{-1}$	$10^3[N]_{free}$ /M	$10^3k_{obs}/s^{-1}$
0.31	17.1	0.30	17.0	0.34	22.2
0.77	44.5	0.76	46.0	0.87	54.4
1.23	79.9	1.22	79.0	1.37	80.0
1.69	113	1.67	104	1.88	109
2.16	134	2.13	142	2.40	150
2.62	175	2.59	148	2.91	191
3.08	200	3.04	205	3.42	236

Table S24. Kinetic data for the reaction of 1-fluor-2,4-dinitrobenzene with Cysteine (Cyst) in water solution at 25ºC±0.1ºC.

Cyst pH=7.8		Cyst pH=8.1		Cyst pH=8.4	
$10^3[N]_{free}$ /M	$10^3k_{obs}/s^{-1}$	$10^3[N]_{free}$ /M	$10^3k_{obs}/s^{-1}$	$10^3[N]_{free}$ /M	$10^3k_{obs}/s^{-1}$
0.76	14.9	1.32	27.9	1.06	19.3
1.90	36.4	3.30	78.2	2.64	50.5
3.04	59.8	5.27	132	4.23	85.5
4.18	95.3	7.25	166	5.81	112
5.32	109	9.23	187	7.40	149
6.46	131	11.2	226	8.98	177
7.60	175	13.2	294	10.6	277

Figure S25. Bronsted plot analysis for ClDNB in water at 25ºC±0.1ºC.

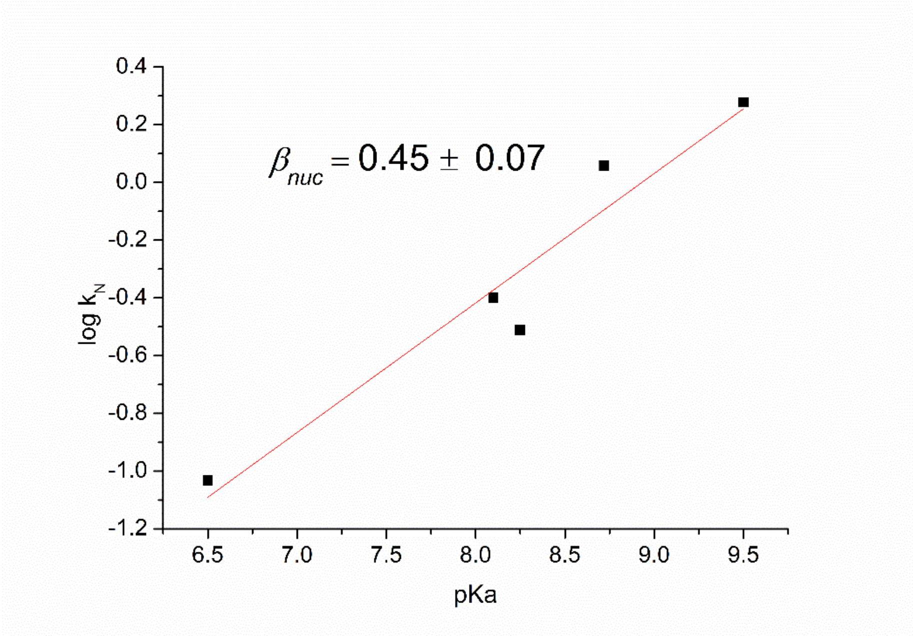

Figure S26. Bronsted plot analysis for ClDNB in water at 37ºC±0.1ºC.

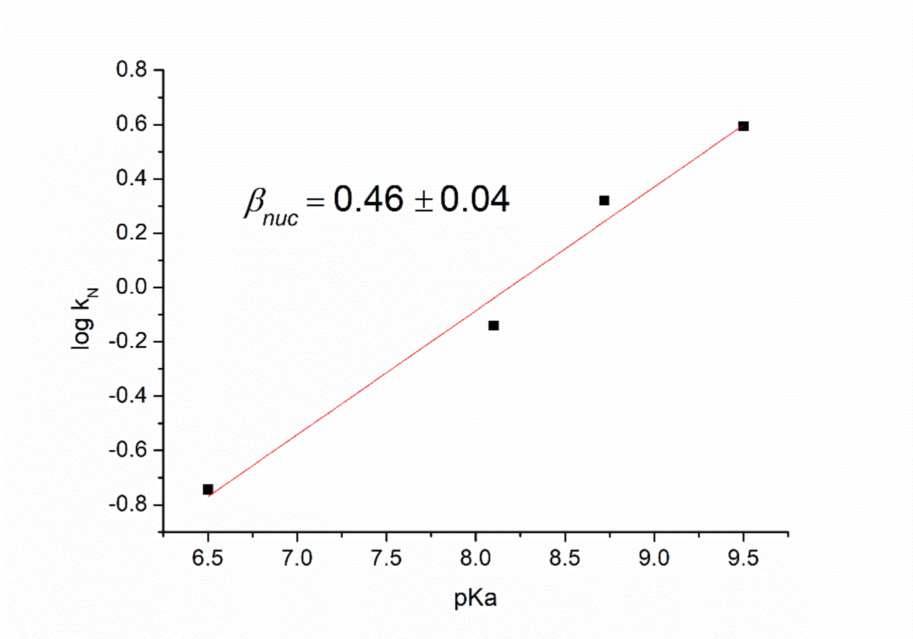

Figure S27. Bronsted plot analysis for ClDNB in buffer phosphate at 25ºC±0.1ºC.

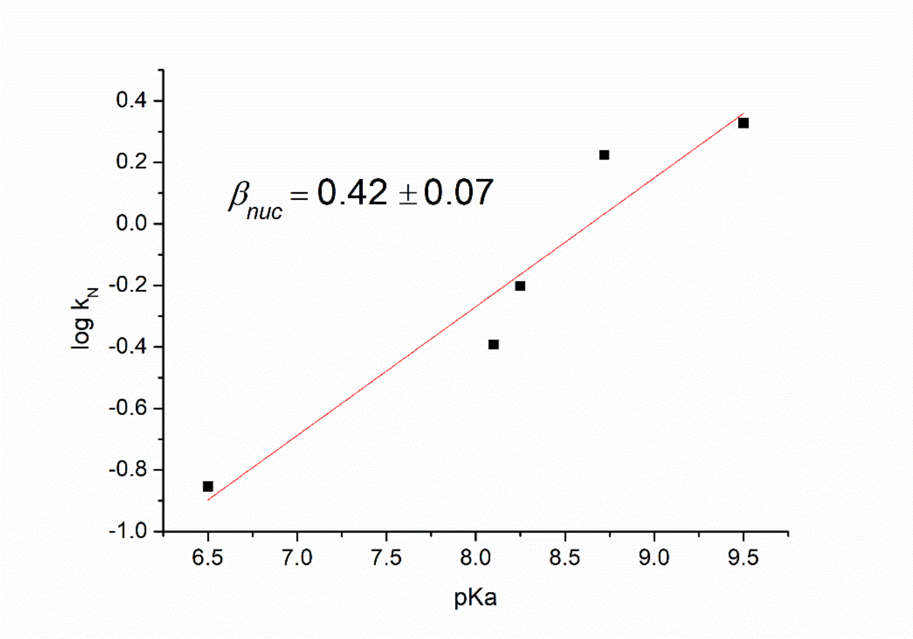

Figure S28. Bronsted plot analysis for ClDNB in buffer phosphate at 25ºC±0.1ºC.

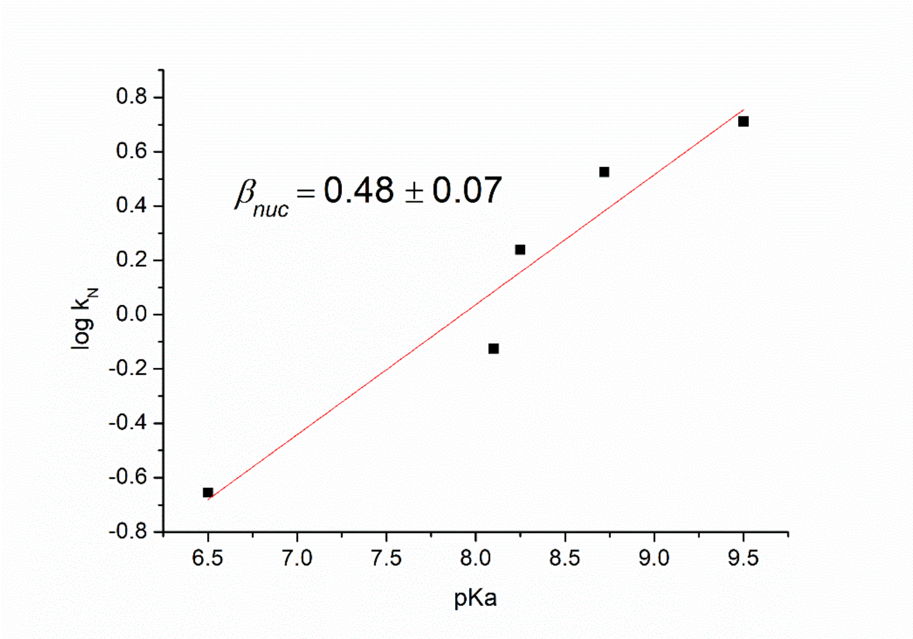

Figure S29. Bronsted plot analysis for FDNB in water at 25ºC±0.1ºC.

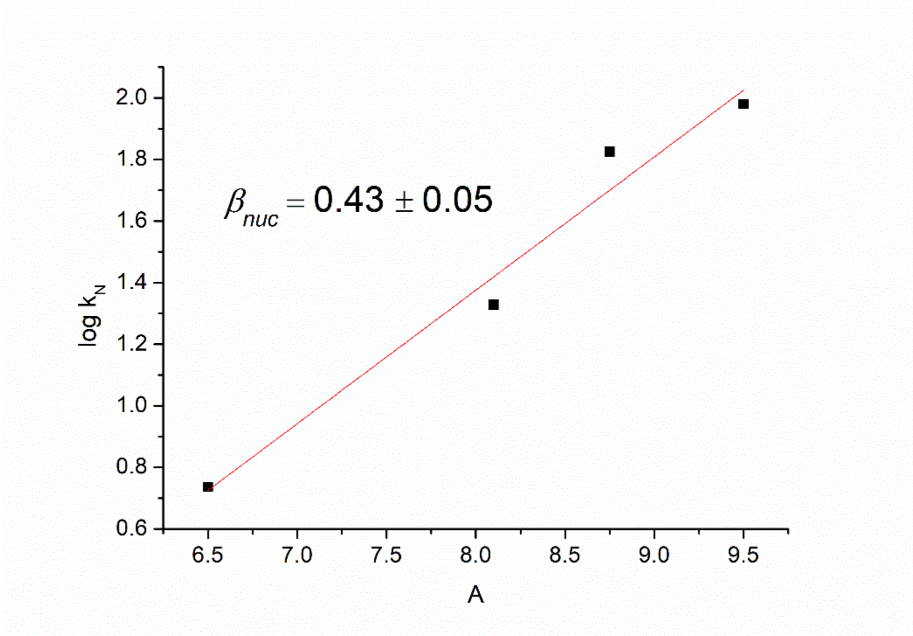

Supplement: Supplementary file 1 [file DataSheet1.PDF]
